# Pigeons (*Columba livia*) as Trainable Observers of Pathology and Radiology Breast Cancer Images

**DOI:** 10.1371/journal.pone.0141357

**Published:** 2015-11-18

**Authors:** Richard M. Levenson, Elizabeth A. Krupinski, Victor M. Navarro, Edward A. Wasserman

**Affiliations:** 1 Department of Pathology and Laboratory Medicine, University of California Davis Medical Center, Sacramento, California, United States of America; 2 Department of Psychological and Brain Sciences, The University of Iowa, Iowa City, Iowa, United States of America; 3 Department of Radiology & Imaging Sciences, College of Medicine, Emory University, Atlanta, Georgia, United States of America; Glasgow University, UNITED KINGDOM

## Abstract

Pathologists and radiologists spend years acquiring and refining their medically essential visual skills, so it is of considerable interest to understand how this process actually unfolds and what image features and properties are critical for accurate diagnostic performance. Key insights into human behavioral tasks can often be obtained by using appropriate animal models. We report here that pigeons (*Columba livia*)—which share many visual system properties with humans—can serve as promising surrogate observers of medical images, a capability not previously documented. The birds proved to have a remarkable ability to distinguish benign from malignant human breast histopathology after training with differential food reinforcement; even more importantly, the pigeons were able to generalize what they had learned when confronted with novel image sets. The birds’ histological accuracy, like that of humans, was modestly affected by the presence or absence of color as well as by degrees of image compression, but these impacts could be ameliorated with further training. Turning to radiology, the birds proved to be similarly capable of detecting cancer-relevant microcalcifications on mammogram images. However, when given a different (and for humans quite difficult) task—namely, classification of suspicious mammographic densities (masses)—the pigeons proved to be capable only of image memorization and were unable to successfully generalize when shown novel examples. The birds’ successes and difficulties suggest that pigeons are well-suited to help us better understand human medical image perception, and may also prove useful in performance assessment and development of medical imaging hardware, image processing, and image analysis tools.

## Introduction

Pathologists and radiologists are confronted daily with perceptual challenges in the medical imaging domain. These individuals, even after years of education and training, may sometimes struggle to arrive at correct disease or risk classifications using the visual cues present on microscope slides or medical images (e.g., x-rays). There is considerable room for enhancing medical image perception and interpretation; this can occur not only via additional visual and verbal training [1–3], but also through ever-improving image acquisition technology, image processing and display. However, such innovations in medical imaging must be validated—using trained observers—in order to monitor quality and reliability. This process, while necessary, can be difficult, time-consuming and expensive. Automated computer-aided substitutes are available, but may fail to faithfully reflect human performance in many cases [4–6]. We describe here a potential alternative approach.

Research over the past 50 years has revealed that pigeons can be prodigious discriminators of complex visual stimuli, and are able to detect or discriminate: foreground from background [7]; misshapen pharmaceutical capsules [8]; letters of the alphabet [9]; basic object categories such as cats, flowers, cars, and chairs [10]; identities and emotional expressions of human faces [11]; and even paintings by Monet vs. Picasso [12], among many other impressive feats. Pigeons’ visual memory is also outstanding, as they can recall more than 1,800 images [13]. Importantly, pigeons have demonstrated an ability to generalize their discrimination performance to novel objects or scenes. We are still a long way from knowing precisely how pigeons so successfully differentiate such complex and varied visual stimuli, but color, size, shape, texture, and configural cues all seem to participate [14–16]. Importantly, however, the anatomical (neural) pathways that are involved, including basal ganglia and pallial-striatal (cortical-striatal in mammals) synapses, appear to be functionally equivalent to those in humans [11].

Given the well-documented visual skills of pigeons, it seemed to us possible that they might also be trained to accurately distinguish *medical* images of clinical significance. We expected that, entirely without verbal instructions, operant conditioning procedures alone could prove sufficient for teaching the birds the intricate visual discrimination skills associated with diagnosing medically relevant images. The basic similarities between vision-system properties of humans and pigeons also suggested that, if pigeons were confronted with pathology and radiology study sets of increasing difficulty, then their task accuracy would track that achievable by domain-expert human observers.

In these initial studies, we sought answers to four questions. First, could discrimination between benign and malignant pathology and radiology images be taught to pigeons—entirely without the benefit of verbal instructions generally provided to human observers? Second, could pigeons go beyond mere memorization and demonstrate generalization (i.e., perform accurately when confronted with related but novel stimuli)? Third, how would pigeons perform when given image discrimination tasks that are extremely difficult even for skilled human observers? And fourth, if the birds were successful, then could such skills have any practical utility?

We provided food reinforcement to pigeon subjects, maintained just below their free-feeding weight, for correctly discriminating—via two distinctively colored response buttons—images of benign or malignant pathology specimens. Initial experiments focused on conventionally stained and digitally scanned breast pathology slides, starting with low (4×) and extending to medium (10×) and high (20×) levels of magnification. The pigeons’ successful mastery of these tasks encouraged us to investigate their ability to detect diagnostically important microcalcifications in mammograms (a challenging, yet tractable task) [17], and also to attempt a much more difficult task, namely to classify benign and malignant breast masses. Correctly identifying these challenging target masses is difficult even for trained radiologists [18]; and, in fact, it proved to lie at the limits of pigeons’ capabilities.

Although pigeons are unlikely to be called upon to offer clinical diagnostic support, it does seem quite possible that their discriminative abilities may be turned to a useful purpose. The need for image quality assessment is a practical one that arises in both pathology and radiology domains. Even though pathologists still largely use optical microscopes for their clinical work, with the recent advent of digital pathology, diagnoses are increasingly being rendered directly from a computer screen rather than through a microscope [19–21]. Image acquisition and digital display technologies—and the accompanying software—are continuously being updated; each technical iteration must be scrutinized to determine the merits and drawbacks of new vs. existing techniques [22]. Such evaluations often require elaborate study designs and, crucially, the participation of one or more skilled observers [23]. However, finding available and affordable clinicians can be difficult at best [24].

This process is similar to what is done in evaluating medical image quality with model or mathematical (so-called “ideal”) observers. The purpose of these software-based tools is not to replicate human performance *per se*, but to help predict, for example, which manipulations of image quality (e.g., color fidelity or compression level) will impact human performance. However, such mathematical models address only narrow sets of questions, are hard to adapt quickly to new problems, and require their own validation *vis a vis* human performance [4–6]. Our research suggests that avian observers might be able to deliver relevant assessments of image diagnostic content and quality, as their performance is reproducible and can easily and quantitatively be tracked by modern conditioning techniques.

## Materials and Methods

### General procedures

#### Subjects and apparatus

Experiment 1 involved a cohort of 8 pigeons (*Columba livia*); Experiments 2 and 3 each involved 4 pigeons. In Experiment 1, 4 pigeons were trained with normal images and the other 4 pigeons were trained with hue- and brightness-balanced monochrome images. In Experiments 2 and 3, all of the training images were of the same type for each of the 4 birds.

All of the pigeons were maintained at 85% of their free-feeding weights by controlled daily rations and were housed in individual cages, with ad-lib access to grit and water. The birds had served in unrelated studies prior to the present project and thus did not require specific pretraining experience. All housing and training procedures were specifically approved by the Institutional Animal Care and Use Committee of The University of Iowa (approval number 1306098).

For counterbalancing purposes, the pigeons in each quartet were divided into two groups (1 and 2) based on which subset of slides they were given during training. Within Groups 1 and 2, the left-right locations of the correct choice responses for malignant and benign breast tissue samples were also counterbalanced.

**Fig 1:**
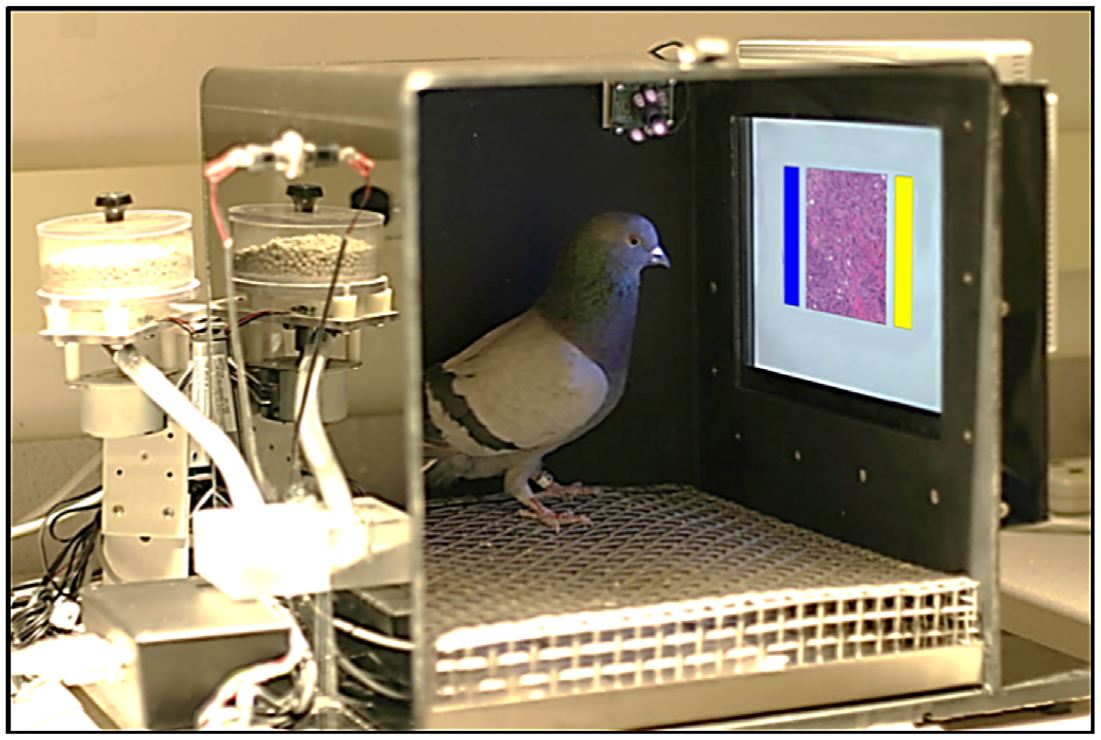
The pigeons’ training environment. The operant conditioning chamber was equipped with a food pellet dispenser, and a touch-sensitive screen upon which the medical image (center) and choice buttons (blue and yellow rectangles) were presented.

The pigeons were trained in operant conditioning chambers. The chambers (shown in Fig 1) measured 36 cm × 36 cm × 41 cm and were located in a dark room with continuous white noise played during sessions. Each chamber was equipped with a 15-in LCD monitor located behind an AccuTouch^®^ resistive touchscreen (Elo TouchSystems, Fremont, CA). The portion of the screen that was viewable by the pigeons was 28.5 cm × 17.0 cm. Pecks to the touchscreen were processed by a serial controller board mounted outside the box. A rotary dispenser delivered 45-mg pigeon pellets through a vinyl tube into a food cup located in the center of the rear wall opposite the touchscreen. Illumination during the experimental sessions remained constant and was provided by a house light mounted on the upper rear wall of the chamber. The pellet dispenser was controlled by a digital I/O interface board attached to an Apple^®^ iMac^®^ computer. Experimental sessions were controlled by a program created and run using MatLab^®^ with Psychtoolbox-3 extensions (http://psychtoolbox.org/) [25, 26].

### Experiment 1. Breast histopathology

#### Standard breast histology stimuli

A total of 144 images (48 at each of three magnification settings) taken from breast tissue samples were used. These images consisted of equal numbers of relatively straightforward benign and malignant examples from routine pathology cases at the University of California Davis Medical Center. They were selected by a pathologist authorized to view the original image files and were anonymized before being shared with the research team. The tissues had been formalin-fixed, paraffin-embedded, sectioned at 5-micron thickness, and stained with hematoxylin and eosin following standard clinical practice. The stained slides were digitally scanned using an Aperio whole-slide scanner at a maximum resolution setting of 20×. Images were then down-sampled to provide representative fields at nominal 4× and 10× resolution. Each image was captured at 388 × 388 pixels, and resized by our program to 308 × 308 pixels; when presented to the birds the images filled an area on the display of 9.1 cm on a side. Additionally, a rotated set of images was created. Each original image (0°) had 90°, 180°, 270°, horizontal flip, and vertical flip versions, yielding a total of 288 (48 × 6) possible exemplars. This set expanded the variety of visual stimuli, and was used during the second part of each training phase to encourage generalization as opposed to memorization.

**Fig 2:**
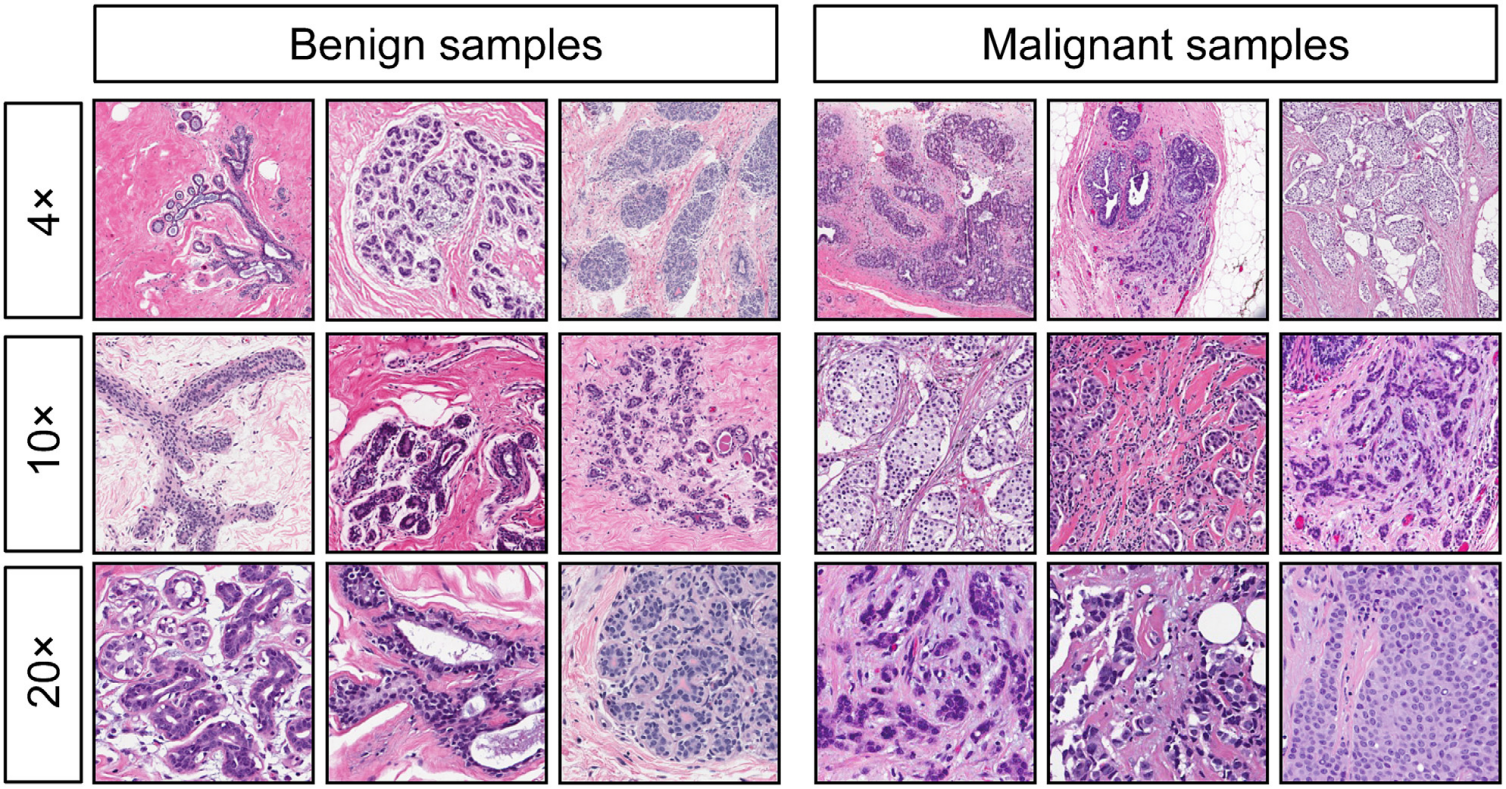
Examples of benign (left) and malignant (right) breast specimens stained with hematoxylin and eosin, at different magnifications. Pigeons were initially trained and tested with samples at 4× magnification (top row), and then were subsequently transitioned to samples at 10× magnification (center row) and 20× magnification (bottom row).

Each magnification grouping was separated into two sets comprising 12 benign and 12 malignant tissue examples (yielding Sets A and B, respectively, for a total of 48 images). Group 1 was trained with Set A and tested with Set B; the opposite occurred for Group 2. See Fig 2 for a representative sample of images displayed to the birds. The complete set is available in the S1 File included in the Supporting Information. Training began with 4× (low-magnification) images, and after a test period in which novel, previously unseen samples were presented to determine how well the birds could generalize, the cycles were repeated with 10× (medium) and 20× (high) magnifications.

#### Training regimen

Daily training sessions comprised 144 trials, on each of which an exemplar from the training set (A or B, depending on the bird) was randomly shown. During the first phase of training, only the 0° orientation of the 24 stimuli was presented; in these sessions, pigeons saw each exemplar 6 times.

At the beginning of each trial, pigeons were presented with a start stimulus: a white square (3 cm × 3 cm) in the middle of the computer screen. After 1 peck within the white square area, a tissue sample was displayed in the center of the screen. The pigeons had to satisfy an observing response requirement within the area of the tissue sample. This requirement began with 1 peck and was adjusted upward in accord with the performance of each pigeon, reaching values from 6 to 10 pecks. If the bird was consistently pecking, but not acquiring the discrimination in a timely fashion, then the number of pecks required was raised to increase the cost of making an incorrect response.

On completion of the observing response requirement, two report buttons appeared 3 cm to the left and right of the tissue sample, as shown in Fig 1. The report buttons were 2.3 cm × 6.0 cm rectangles filled with either a solid blue (RGB 0, 100, 242) or yellow color (RGB 251, 249, 23). Pigeons had to select one of the two report buttons depending on the kind of tissue sample presented (benign or malignant) and the counterbalanced correct response assignment. If the choice response was correct, then food reinforcement was delivered and an intertrial interval (ITI) that randomly ranged from 6 to 10 s ensued. If the choice response was incorrect, then food was not delivered and a correction trial with the same exemplar was given after the ITI. Correction trials were given until the correct response was made. No data were analyzed from correction trials.

After an initial training period of 15 days using stimuli presented in a single (0°) orientation, all pigeons received a second phase of training, in which all six orientations of the 24 stimuli in a given set were presented; in these sessions, pigeons saw each exemplar only once. Training sessions with this set were given over 5 consecutive days, and the birds were next moved to novel stimulus testing.

#### Test regimen with novel stimuli

Each testing session comprised the original 144 training trials with the rotated set of stimuli plus 24 testing trials in which novel exemplars (i.e., stimuli not seen during the training phase) were presented, for a total of 168 trials. These novel exemplars consisted of Set B if pigeons had been trained with Set A, and Set A if pigeons had been trained with Set B. Birds were exposed to these novel tissue exemplars only during testing trials and they saw each testing exemplar only once daily. Testing trials also differed from training trials in that they were nondifferentially reinforced, as follows: On *training* trials, only the correct response was reinforced; incorrect responses were followed by correction trials (differential reinforcement). On *testing* trials, any choice response was reinforced (nondifferential reinforcement); food was given regardless of the pigeons’ responses, so that testing could proceed without teaching the birds the correct responses to the testing exemplars. On testing trials, the designations of “correct” or “incorrect” for choice responses were for scoring purposes only, as no correction trials were necessary. Testing sessions were given over 5 consecutive days.

#### Novel magnification testing

The second phase of testing utilized tissue sample exemplars at higher magnifications; after training at 4× magnification, the birds were exposed to 10× magnification images. Novel magnification testing trials were also nondifferentially reinforced, with food given regardless of the pigeons’ choice response to preclude the birds’ learning about the testing exemplars. The 24 testing trials were again intermixed with 144 differentially reinforced training trials. Testing sessions at novel magnifications were given over a period of 5 days.

After this phase of testing, the same birds started the training regimen with images at 10× magnification and were tested with novel and then higher magnification stimuli as previously described. Subsequently, they repeated the same cycle with images at 20× magnification, except this time no higher magnification testing was given.

#### Monochrome images with normalized hue and luminance

Results from full color image training and testing established that pigeons could adeptly discriminate malignant from benign breast histological samples at multiple levels of magnification, in different spatial orientations, and effectively transfer those discriminations to novel stimuli. Not yet determined was what properties of the tissue samples enabled the pigeons to perform so accurately. Therefore, we sought to eliminate color and luminance cues to limit the range of features available for discrimination, and also studied the effects of different levels of image compression on accuracy, compressing the images (using the JPEG standard) to 7% and 4% of their original size. We monitored the pigeons’ discrimination of all of these modified images to see whether and to what degree performance was affected.

#### Monochrome stimuli

**Fig 3:**
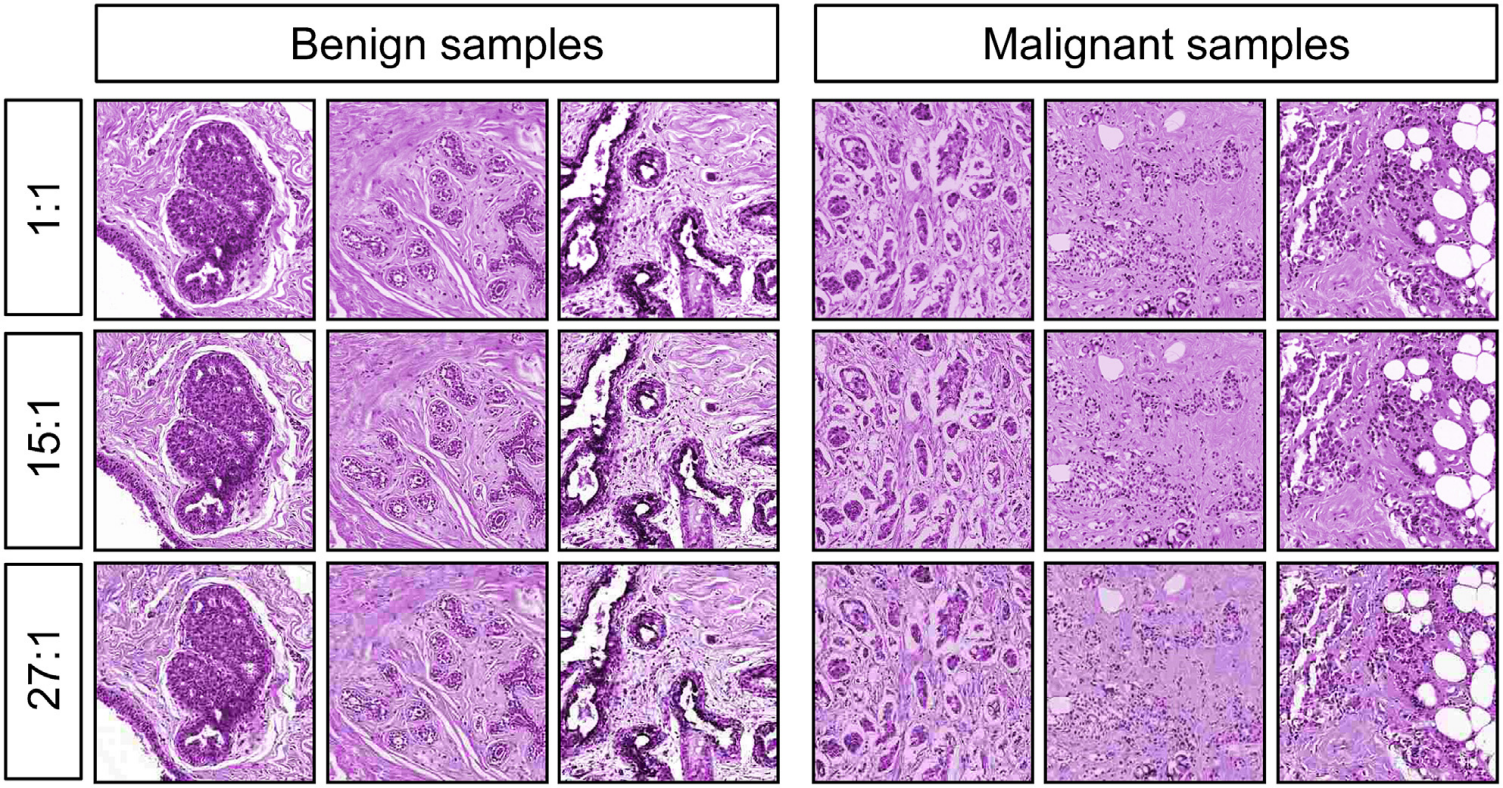
Monochrome images with equated hue and brightness, at different levels of compression. The original images at 10× magnification were converted to grayscale, colored with a single hue, and had their overall brightness and contrast equalized as closely as possible. Additionally, the images were reduced to 7% (1:15, middle row) or 4% (1:27, bottom row) of their original size, to create the compressed sets.

The 10× stimuli at 0° were used, but were converted to monochrome and equated in hue and brightness to eliminate those image properties as variables (see Fig 3, top row, for representative images). Using GIMP (www.gimp.org), images were converted from RGB to grayscale, and then pseudocolored to a hue (290) that was approximately the average hue of the full-color H&E images. As every pixel was represented by the same hue (but with variable intensity values), no sample-specific color information was being conveyed. After re-coloring, the overall brightness and contrast levels were manually adjusted to minimize differences between cancer and normal samples. The complete set is available in the S1 File included in the Supporting Information.

#### Monochrome training and testing; per-bird and cohort voting scores

Four different pigeons received initial training (15 days), rotation training (5 days), and novel stimulus testing (5 days) just as with the phases used during presentation of the standard breast histology stimuli. Thereafter, the pigeons were trained with both the familiar and novel sets of stimuli at the 0° orientation on alternate days, receiving 144 differentially reinforced trials each day. Following that alternation training, similar daily sessions with all 48 stimuli intermixed were given for 9 days.

Finally, full-set testing was performed using monochrome images in which all birds saw all of the images (10×, n = 48). Accuracy was scored on a per-bird basis as well as through a group-voting scheme that we termed “flock sourcing.”

#### Compressed image stimuli

The same monochrome images described above were presented to the birds along with additional versions that had been compressed to 7% (15:1) and 4% (27:1) of their original size (1:1). Compression was performed using the open-source Caesium package at its nominal 30% and 90% compression settings (http://caesium.sourceforge.net/) using libJPEG (http://libjpeg.sourceforge.net/), embedded in the Qt5 framework (http://qt-project.org/qt5), and saved in JPEG format. Representative examples are shown in Fig 3, rows 2 and 3. The complete set is available in the S1 File included in the Supporting Information.

#### Compressed image testing and training

To accommodate the large number of testing stimuli (original and compressed images), testing was divided into 4 days; each day involved 144 trials with the complete set of original stimuli under differential reinforcement intermixed with 24 trials with compressed stimuli (6 per category at the two different compressions) under nondifferential reinforcement. A total of 6 testing cycles were completed, for a total of 24 days.

Subsequently, over 15 daily sessions, both the original and compressed stimuli were trained under differential reinforcement. Each daily session involved 48 trials with the compressed stimuli intermixed with 96 trials with the original stimuli, yielding a total of 144 trials.

### Experiment 2. Microcalcifications in mammograms

**Fig 4:**
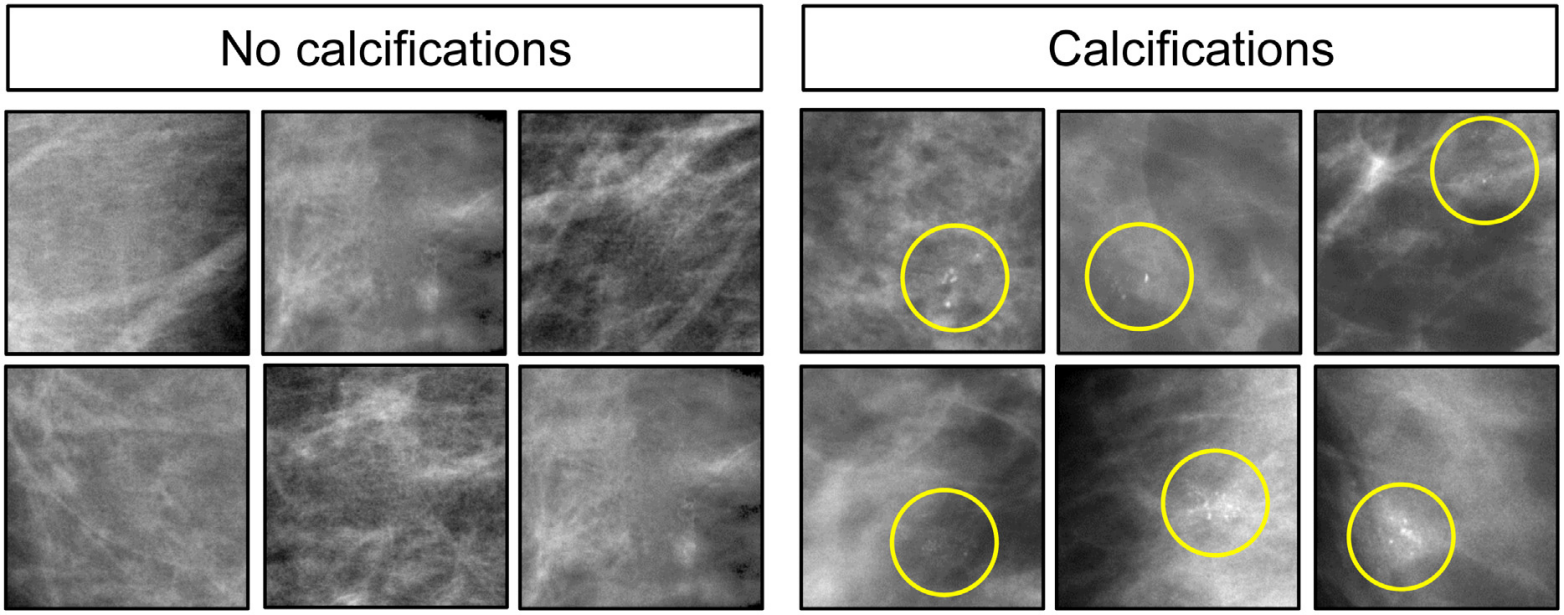
Mammograms with the absence (left) and with presence (right) of microcalcifications. Yellow circles denote where microcalcifications are located.

Switching from the domain of pathology to that of radiology, we explored pigeons’ visual capabilities with a pair of breast-cancer-relevant mammogram-based studies. In the first series, using a similar training and testing regimen as described for the histopathology studies, a new cohort of birds was exposed to regions of mammograms devoid of or containing subtle microcalcifications (Fig 4). These often-difficult-to-discern calcium deposits have considerable radiographic significance, as they can be reliably associated with the presence of cancer [17].

#### Stimuli

A total of 40 regions of interest were cropped from anonymized mammograms approved for research use by the University of Arizona IRB: 20 containing subtle clusters of microcalcifications plus 20 examples without clusters (see Fig 4 for representative images; a complete image set is available in the S2 File included in the Supporting Information). Each image had original dimension of 256 × 256 pixels, and after resizing measured 308 × 308 pixels. The images were split into two sets (A and B) consisting of 10 calcification and 10 no-calcification exemplars. These images had been previously shown (by EAK) to 10 radiologists (5 experienced mammographers and 5 senior-level radiology residents) in a study designed to test their ability to detect microcalcifications. Average human accuracy scores were used to partition the images shown to the birds across the two sets to ensure that each set was approximately equal in terms of (human) difficulty levels. Brightness and contrast values were modestly adjusted by hand to ensure that these values were approximately equivalent across all images. Additionally, a set of rotated images was created by applying to each stimulus the same rotations and flips as described in Experiment 1.

#### Training and testing

Daily training sessions comprised 120 trials during which an exemplar from either Set A (for 2 birds) or Set B (for the other 2 birds) was randomly shown. Initially, only the 0° orientation of the 20 stimuli was presented; in these training sessions, pigeons saw each exemplar six times. Later, all six orientations of the 20 stimuli were presented; in these sessions, pigeons saw each exemplar only once. The training regime was like that described for Experiment 1.

Birds were trained with the stimuli in their original 0° rotation alone for a total of 25 days, at which point all of the birds were accurately categorizing the calcification and non-calcification stimuli. After the initial training period, a full set of rotated stimuli was introduced and birds were given an additional of 15 days of full-rotation training. All birds quickly reached high levels of discrimination accuracy with this expanded stimulus set and were moved next to novel stimulus testing.

Each novel stimulus testing session comprised 120 training trials and 20 testing trials, randomly presented, for a total of 140 trials. During testing, birds were shown novel exemplars consisting of images from the opposing set of stimuli that birds had seen during their training. Birds were exposed to these novel exemplars only during testing trials, and saw each testing exemplar only once. As before, testing trials differed from training trials in that they were nondifferentially reinforced. On such testing trials, the designations of “correct” or “incorrect” choice responses were for scoring purposes only, as no correction trials were necessary. Testing sessions were given over 5 consecutive days.

### Experiment 3. Mammogram masses

The second radiology study involved mammogram images containing (microcalcification-free) masses, with the image sets evenly split between benign or malignant as determined upon subsequent biopsy evaluation.

#### Stimuli

**Fig 5:**
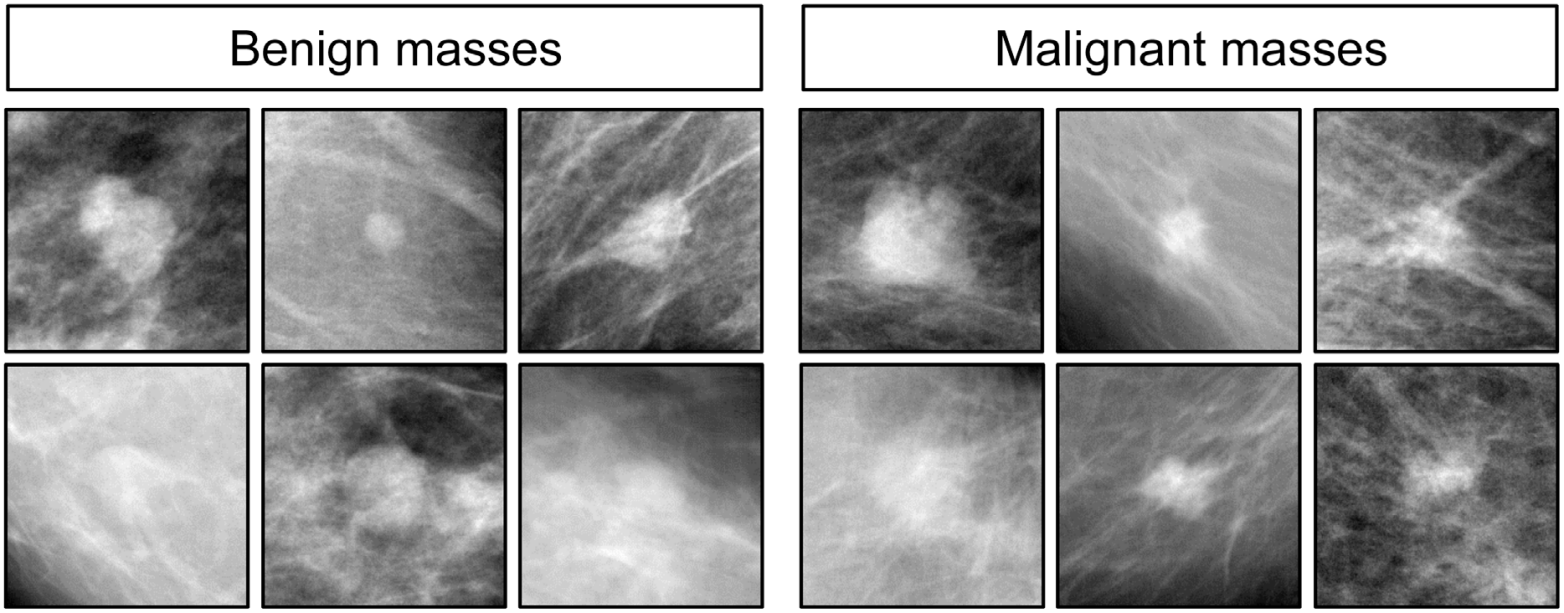
Examples of benign (left) and malignant (right) masses in mammograms. Subsequent biopsy established histopathology ground-truth.

A total of 40 region-of-interest images cropped from anonymized mammograms approved for research use by the University of Arizona IRB, consisting of 20 samples with malignant masses and 20 samples with benign masses were used (see Fig 5 for representative images). The complete set is available in the S2 File included in the Supporting Information. Each image presented to the birds measured 308 × 308 pixels. These images had been previously shown (by EAK) to 6 radiologists (3 experienced mammographers and 3 senior-level radiology residents) in a study designed to test their ability to detect and evaluate masses. The images were split into two sets, each consisting of 10 malignant and 10 benign masses (yielding Sets A and B, respectively). The two sets were again equated for brightness and human difficulty ratings, as described for the microcalcification stimuli. Additionally, a set of rotated images was created by applying to each stimulus the same rotations and flips as in Experiment 1.

#### Training and testing

Daily training sessions comprised 120 trials on each of which an exemplar from either Set A or Set B was randomly shown, and unlike the previous experiments, all six orientations of the 20 stimuli were presented from the outset; pigeons thus saw each exemplar only once in each session. Training and testing was performed as described in Experiment 2. After the birds had reached asymptotic levels of discrimination accuracy (80 days; N.B., much longer than needed for previous tasks), they were moved directly to testing.

#### Statistical analyses

Analyses across all experiments were performed by calculating individual accuracy scores and submitting them to repeated-measures ANOVA (Analysis of Variance) using SPSS^®^ version 21. The ROC analysis was performed with R (http://cran.r-project.org/) version 3.1.3, using the pROC package [27], version 1.8. The alpha level was set to 0.05. When pairwise comparisons were used to follow up main effects with more than two levels, p-values were adjusted using the Dunn–Šidák correction for multiple testing. In the Results section below, all statements asserting statistical significance reflect p-values less than or equal to 0.05, and error bars represent the standard error of the mean. Individual bird experimental data are provided in the S3 file.

## Results

### Experiment 1: Full-color histopathology

**Fig 6:**
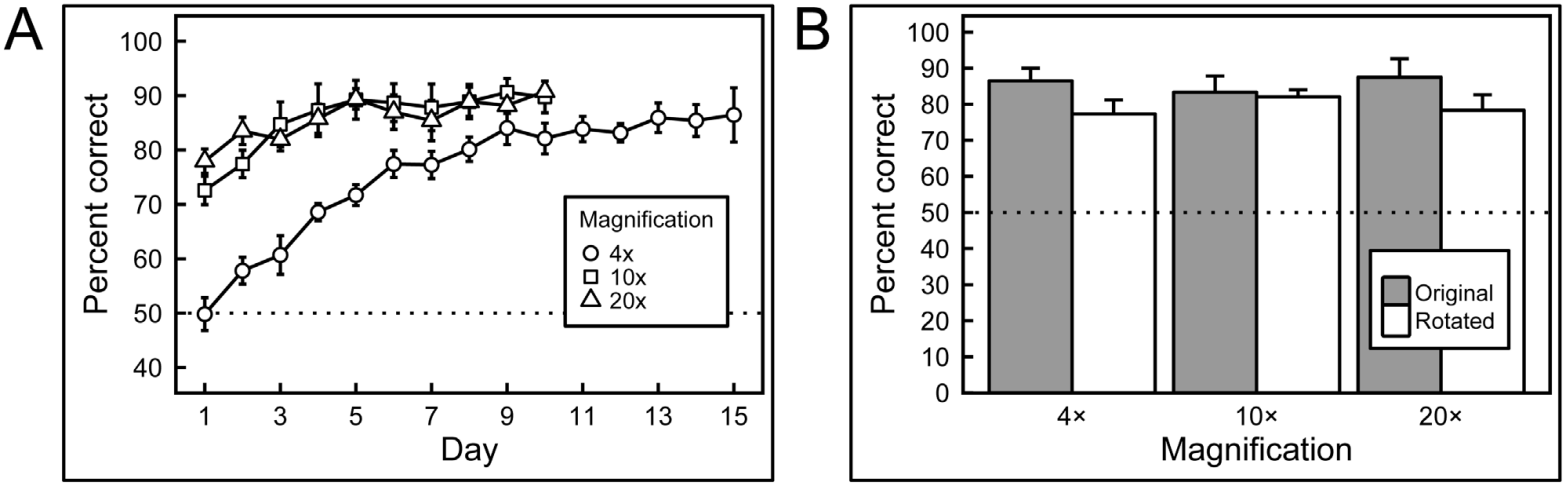
Results of training with breast histopathology samples at different magnifications and rotations. A) When first trained with 4× magnification images the birds performed at chance levels of accuracy, but quickly learned to discriminate. Subsequently, when the birds were exposed to higher magnifications samples, their performance commenced at accuracies above chance (but below their final performance at lower magnification they had previously been exposed to), and improved further with training. B) Introducing rotated versions of the training stimuli did not significantly affect performance at any of the magnifications.

Remarkably, the pigeons rapidly learned to discriminate the appearance of benign from malignant breast tissue histology with high accuracy (Fig 6A), correct choice responses levels rising from 50% at the outset (i.e., at chance level) to 85% over 15 days of training. That rise was statistically significant, *p* = 0.001. Over the succeeding 5 days, pigeons were trained with all rotations of the original image set. Fig 6B illustrates that, on Day 1 of rotation testing, accuracy to the original 0° slides averaged 86% correct, whereas accuracy to the five newly rotated slide views averaged 77% correct, a modest, but non-significant difference, *p* = 0.161; in each case, accuracy exceeded that expected by chance, one-tailed binomial tests, *p* < 0.001. Over the ensuing 4 days, accuracy to the 0° and rotated views converged, averaging 88% correct and 83% correct, respectively, on the fifth day of training (not shown).

#### Novel stimulus testing: memorization or learning?

**Fig 7:**
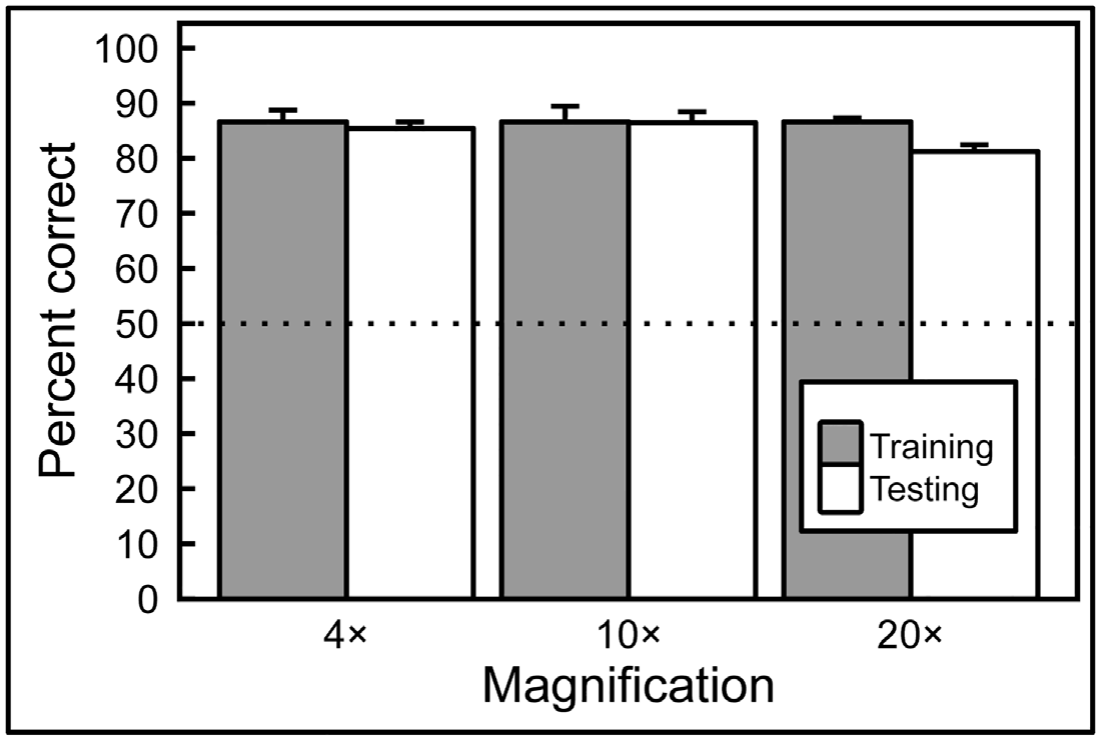
Generalization from training to test image sets. After training with differential reinforcement, the birds successfully classified previously unseen breast tissue images in the testing sets, at all magnifications, with no statistically significant decrease in accuracy compared to training-set performance.

While the birds readily succeeded with this classification task, it was crucial to determine whether they were relying on just their ability to memorize image classification status, or whether they had managed to detected feature-based cues that allowed them to accurately classify previously unseen, novel, images. Accordingly, during a 5-day period after the end of training at each magnification level, pigeons were given a small number of novel benign and malignant breast tissue images intermixed with the full set of familiar training images. Fig 7 shows indeed that the birds had gained the ability to accurately classify novel as well as familiar benign and malignant images, and with equal accuracy, averaging 87% and 85% correct on familiar and novel examples, respectively, a non-significant difference. In each case, accuracy exceeded that expected by chance, one-tailed binomial tests, *p* < 0.001. The pattern of responding to the familiar and novel slides over the next 4 days of non-differentially reinforced testing was similar to that seen on Day 1, averaging 85% and 83% correct, respectively (not shown). Moreover, the birds demonstrated equal learning prowess at all magnifications tested (Fig 7).

Overall, the results show that pigeons were capable of accurately discriminating between benign and malignant breast tissue samples at different levels of magnification, and that they could reliably transfer that performance to novel tissue samples at the same level of magnification. While rotating previously learned images, or presenting similar images, but at higher magnifications than the birds had been trained with, did monetarily affect accuracy, the birds were capable of overcoming these immediate negative effects with the help of additional differential reinforcement-based training.

#### Normalized hue and luminance

**Fig 8:**
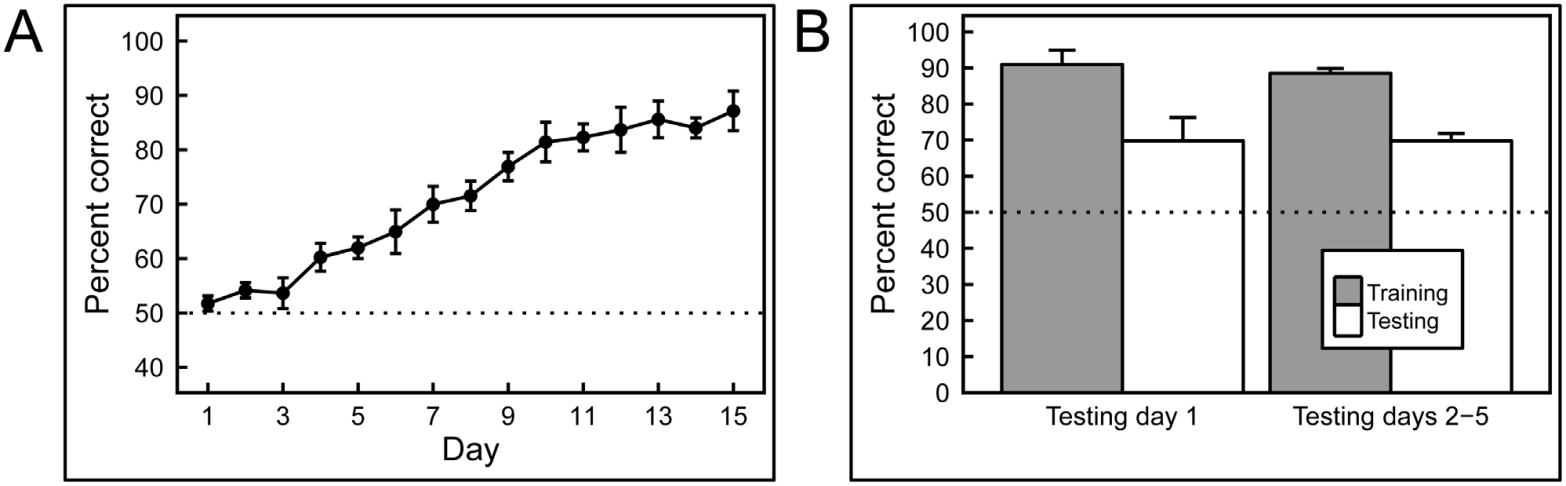
Training and testing with hue- and brightness-normalized breast histology images. A) The pigeons were able to learn discrimination without the benefit of hue and brightness cues. B) However, the lack of these cues diminished the birds’ ability to generalize to new images; compared to an equivalent test of full-color exemplars (see Fig 7), the pigeons performed significantly more poorly, although still well above chance levels.

With these avian accomplishments in hand, we sought to determine how modifying important aspects associated with current aspects of image acquisition and storage—namely, color, brightness, and compression artifacts—might affect the birds’ performance. Pigeons were exposed to monochrome, hue-normalized benign and malignant breast images at 10× magnification and achieved high levels of accuracy over 15 days of training (Fig 8A), which again proved to be unaffected during image rotation trials (not shown). However, as Fig 8B illustrates, when birds were shown to novel monochrome images, their accuracy, while still above chance, was inferior to that when they viewed novel full-color test images (compare Fig 7 with Fig 8B).

#### Full-set monochrome training and “flock sourcing”

Up to this point, we have been reporting accuracy on an averaged per-bird basis, but we wondered whether we could harness the wisdom of crowds, or in this case, a set of 4 birds, to improve the accuracy of avian classification. We examined this possibility after training all 4 birds with the complete set of 10× monochrome images (i.e., Set A plus Set B, n = 48). The mean per-bird percentage of correct choice responses rose significantly from 90% to over 95% correct for the training sets and from 74% to 96% correct for the originally novel stimulus sets across 30 days of training, with alternating familiar and novel training sets. Over the following 9 days, the pigeons were trained with the full set of stimuli intermixed in individual sessions.

Using the first day’s data, we then calculated a “flock score” to represent group performance, and compared it to the individual birds’ performance using a Receiver Operating Characteristic (ROC) analysis. The flock score for a given stimulus was equal to the sum of ‘cancer’ judgments made by the birds. So, if 1 out of the 4 birds made a ‘malignant’ judgment when presented with a tissue sample, then the flock-sourcing score for this stimulus was 1; and, if 3 out of the 4 birds made a ‘malignant’ judgment when presented with a tissue sample, then the flock-sourcing score for this stimulus was 3. Additionally, we considered the real categories of the exemplars as the different states: ‘malignant’ was the positive state (1) and ‘normal’ was the negative state (0).

**Fig 9:**
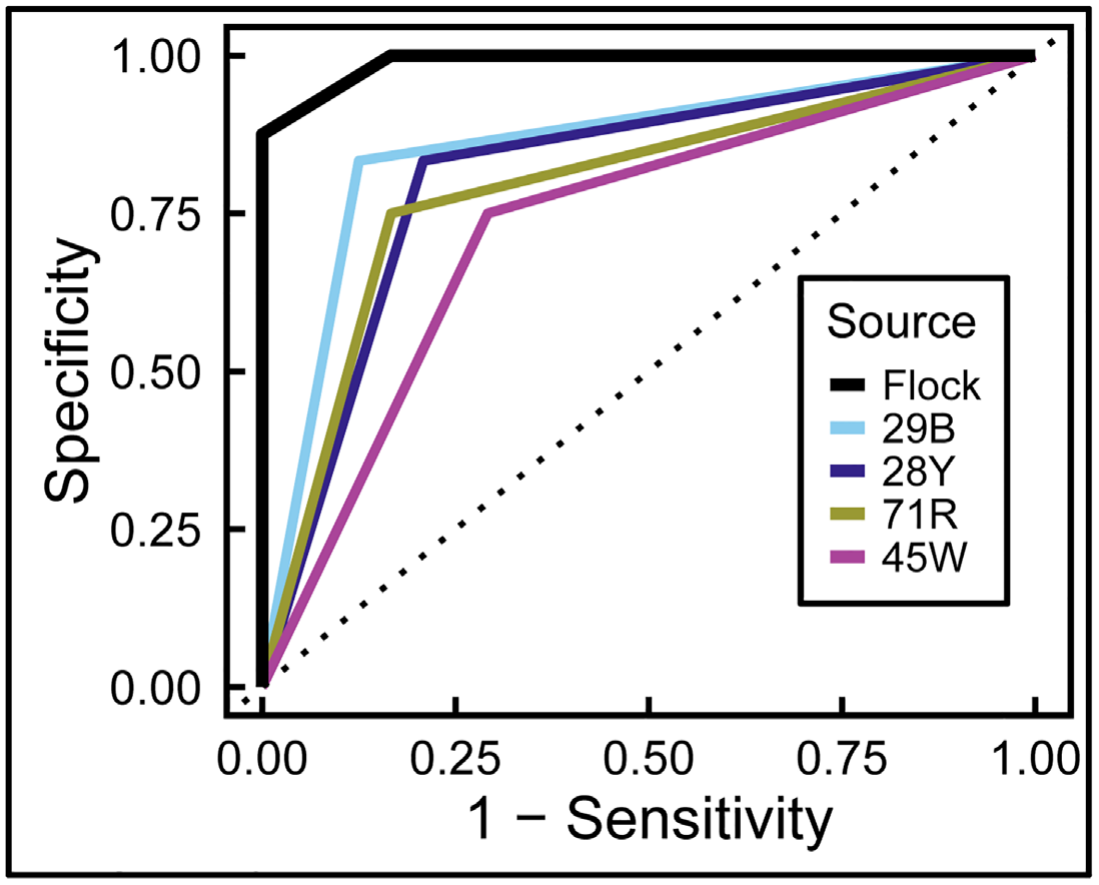
Flock sourcing. A “flock-sourcing” score was calculated by summating the responses of individual birds as described in the text. Pooling the birds’ decisions led to significantly better discrimination than that achieved by individual pigeons. The dotted line represents no discrimination between benign and malignant exemplars.

As Fig 9 shows, even though every bird discriminated well above chance level (areas under the curve: 0.85, 0.81, 0.79, 0.73 for individual pigeons, significantly different from chance), individual bird performance was surpassed by the flock score; indeed, the area under the curve for the flock was 0.99. Most notably, the group’s discrimination proved to be reliably different from that of every individual bird (all *p* values < .01).

#### Effects of image compression

**Fig 10:**
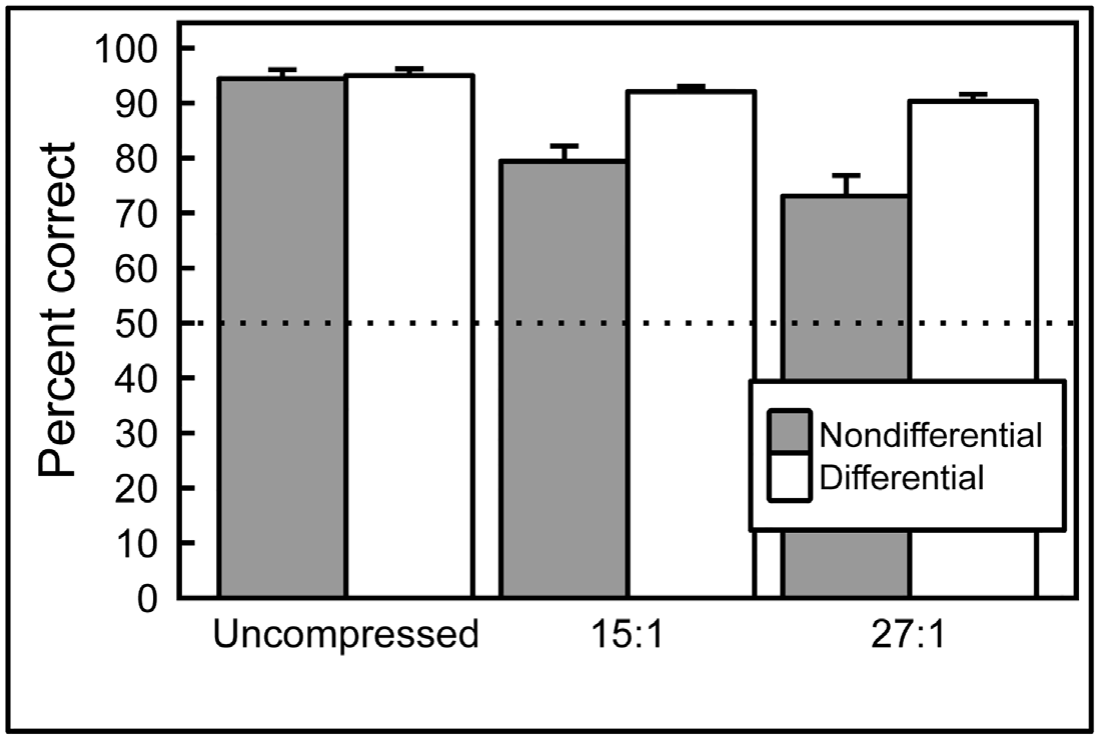
Effect of JPEG image compression. When correct/incorrect responses were nondifferentially reinforced (gray bars), pigeons’ accuracy was affected proportionally to the compression level of the images shown. However, pigeons were capable of achieving high levels of accuracy with compressed images if feedback for correct/incorrect responses was given (white bars).

The cohort of pigeons trained on the 10× monochrome images was then exposed to the same 10× images that had been compressed at two different compression levels (see Fig 3). These compressed images were shown to the birds, initially without differential reinforcement, intermixed with the original uncompressed training set. As Fig 10 shows (gray bars), responses to the uncompressed, 15:1 compression, and 27:1 compression slides across 6 cycles of testing revealed an impact of compression level, with accuracies averaging 94%, 79%, and 73% correct, respectively; pairwise comparisons revealed reliable differences among all three levels of compression (all *p* values < .05).

However, when differential reinforcement was introduced for all compressions levels, over the following 15 days, accuracy rates for all settings converged. Performance with uncompressed slides averaged 95% correct; the 15:1 and 27:1 compression slides averaged 92% and 90% correct, respectively (see Fig 10, white bars). Pairwise comparisons revealed reliable differences between uncompressed and 15:1 (*p* = .017), and between uncompressed and 27:1 (*p* = .005) compression levels, but no significant difference between the two compression levels (*p* = .060).

Overall, these studies demonstrate that image manipulations can reliably affect pigeons’ discrimination performance. Removing luminance and wavelength cues from the images did not measurably impact discrimination learning, although it reduced (but did not eliminate) discriminative transfer (generalization) to new images. Also, image compression deleteriously affected pigeons’ discrimination performance when those compressed images were suddenly introduced and responses to them were nondifferentially reinforced. Yet, pigeons were capable of discriminating those compressed stimuli at high levels of accuracy after further (differentially reinforced) training, although accuracy was still a bit lower than to the original stimuli.

### Experiment 2: Mammograms with microcalcifications

**Fig 11:**
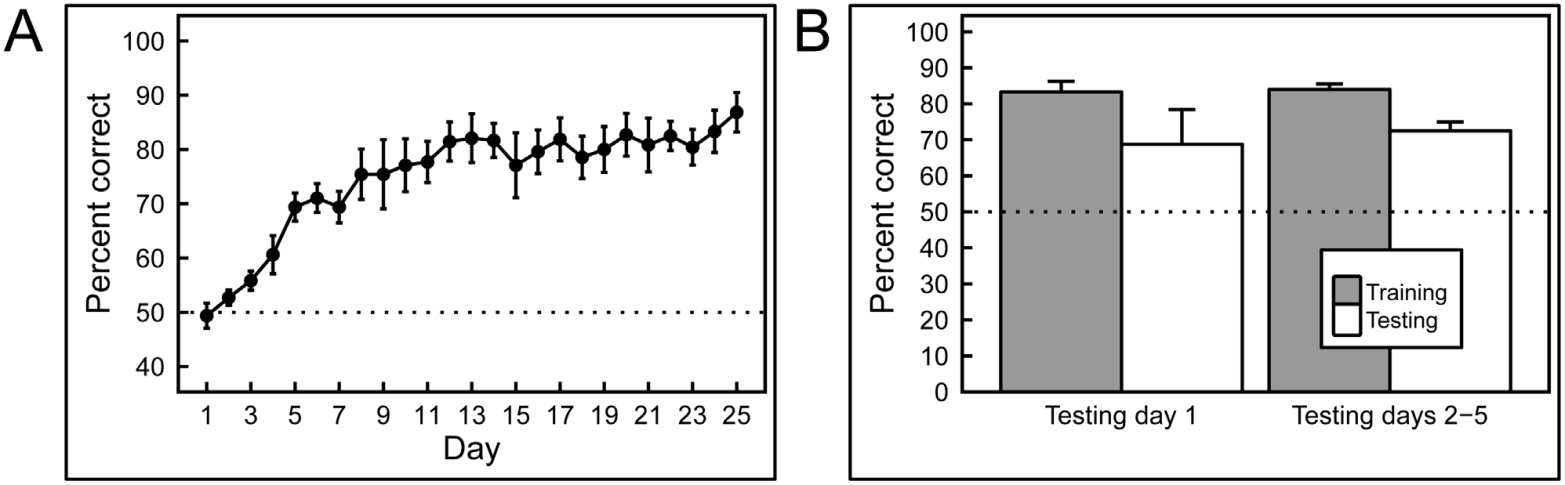
Results of training and testing with mammograms with or without calcifications. A) Training quickly led to high levels of accuracy. B) The pigeons were able to generalize to novel images, but their performance on this task was not as good as their generalization to novel histology images (Fig 7), although still above chance levels of responding. The trend observed on Day 1 of testing (left) continued throughout the remainder of testing (right).

Birds were able to learn to classify images with and without clusters of microcalcifications as adeptly as they had mastered the initial histopathology challenge. Fig 11A shows that accuracy scores rose significantly from 50% to over 85% with 14 days of training, and the addition of rotation and image flipping only briefly and non-significantly affected accuracy. With additional training, scores for the un-rotated and rotated views converged, averaging 86% and 82% correct, respectively.

Over the next 5-day period, pigeons were given a small number of novel images with and without microcalcifications. Accuracy classifying the familiar images was greater than that for the novel images on the first day of testing, averaging 83% correct and 69% correct, respectively, but this difference was not statistically significant. This pattern persisted over the next 4 days of testing, with accuracy averaging 84% correct and 72% correct, for familiar and novel images respectively, both significantly exceeding the level expected by chance (Fig 11B). In a study of human radiologists and radiology residents given the same cases to review, accuracy averaged 97% for calc-(negative) images, but only 70% for calc-(positive) images; our birds’ performance did not reliably differ between the categories. In fairness, unlike the pigeons, the radiologists were not shown just small regions of interest, but whole images that had to be searched for abnormalities, a more challenging task [28].

### Experiment 3: Mammograms with masses

**Fig 12:**
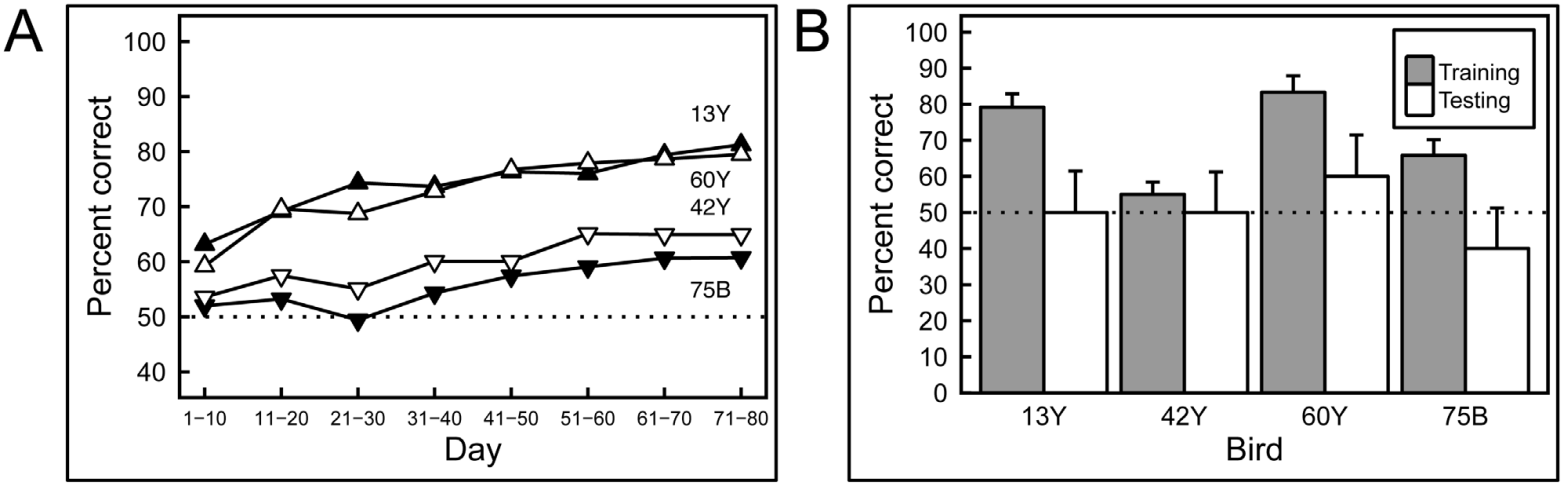
Results of training and testing with mammograms containing masses. A) Pigeons required long training to discriminate between mammograms with masses, and even then, individual differences were pronounced. B) Regardless of their performance in the training phase, all of the pigeons failed to transfer their performance to novel exemplars, suggesting that their performance was based on rote memorization.

As can be appreciated by scrutinizing the representative images depicted in Fig 5, discriminating benign from malignant masses is very challenging indeed; the distinguishing features are extremely subtle. The birds found this to be a much harder task than the others described here. First, it took weeks rather than days for the birds to demonstrate successful learning of the training-set images, and, for the first time, there were notable disparities in the discrimination scores of the individual pigeons. Fig 12A shows that the mean percentage of correct choice responses of two pairs of pigeons respectively rose to around 80% and 60% over 8 blocks of 10 days of training (almost 12 weeks), and one bird never managed to achieve statistically greater-than-chance performance.

Strikingly, when the birds were subsequently tested with novel images, they utterly failed to exhibit significant generalization. Day 1 performance (Fig 12B) during testing yielded average accuracy scores across all birds for familiar and novel images of 71% and 50% correct, respectively, with the scores averaging 74% and 44% respectively over the next 4 days. We conclude that the birds’ modest (albeit slowly achieved) performance during the training phase reflected successful image *memorization*, because despite exhibiting clear discrimination learning, even the two most accurate pigeons displayed no reliable *generalization* to the testing stimuli.

## Discussion

### Histopathology: benign vs. malignant classification

We found pigeons to be remarkably adept at several medical image classification tasks. They quickly learned to distinguish benign from malignant breast cancer histopathology (Fig 2) at all magnifications, a task that can perplex inexperienced human observers who typically require considerable training to attain mastery, although the examples shown are easily classified by a trained pathologist. Starting with the low-magnification (4×) examples, pigeons’ accuracy increased from 50% correct (i.e., chance) on Day 1 to near 85% by Days 13–15 (Fig 6A). There was no notable difference in final accuracy levels on the 4×, 10×, and 20× images sets, indicating the birds could exploit image features at multiple spatial scales. Being trained at one magnification primed the birds for above-chance performance at the next magnification. To ensure that the pigeons were not simply relying on rote memorization, performance was assessed on a testing set comprising new images presented to the birds without differential food reinforcement.

Remarkably, the pigeons were able to distinguish benign from malignant breast histopathology virtually as well on test image sets as they did on the training sets, at all magnifications Fig 7), indicating that they had somehow learned to detect critical discriminating features. The pigeons proved to have an affinity for histopathology, as they achieved stable high performance as fast as or faster than with other visual discrimination problems studied in our Iowa laboratory. Moreover, if instead of scoring per-bird performance, a “flock-sourcing” approach was taken, in which the birds in essence voted, then even higher levels of accuracy could be achieved: when a cohort of 4 birds was shown the full set of uncompressed images, the resulting “group” accuracy level reached an amazing 99%.

Benign and malignant breast histopathology images can differ in terms of overall hue and brightness due to variations in cellular content and tinctorial properties. To ensure that these relatively trivial (but systematically present) differences were not solely responsible for the birds’ classification accuracy, we removed color cues from the same training and testing sets used previously, and explored the effect on pigeons’ learning and transfer behavior. The images were converted from full-color to monochrome, and then pseudo-colored with a single purplish hue that was the same as the average hue of the full-color scanned specimens. In addition, because there was a trend for the cancer samples to be darker and to have higher contrast than their benign counterparts, we manually adjusted overall brightness and contrast levels to minimize differences between the benign and malignant image sets (Fig 3, top row). These two modifications were designed to emphasize the impact of variations in tissue morphology (or texture) on discrimination behavior, while minimizing the roles that color or staining intensities might play. When a new cohort of pigeons was exposed to these monochrome, brightness-normalized images, they were still able to learn and generalize, indicating that morphology and/or texture differences alone were sufficient to sustain classification accuracy (but see below).

**Fig 13:**
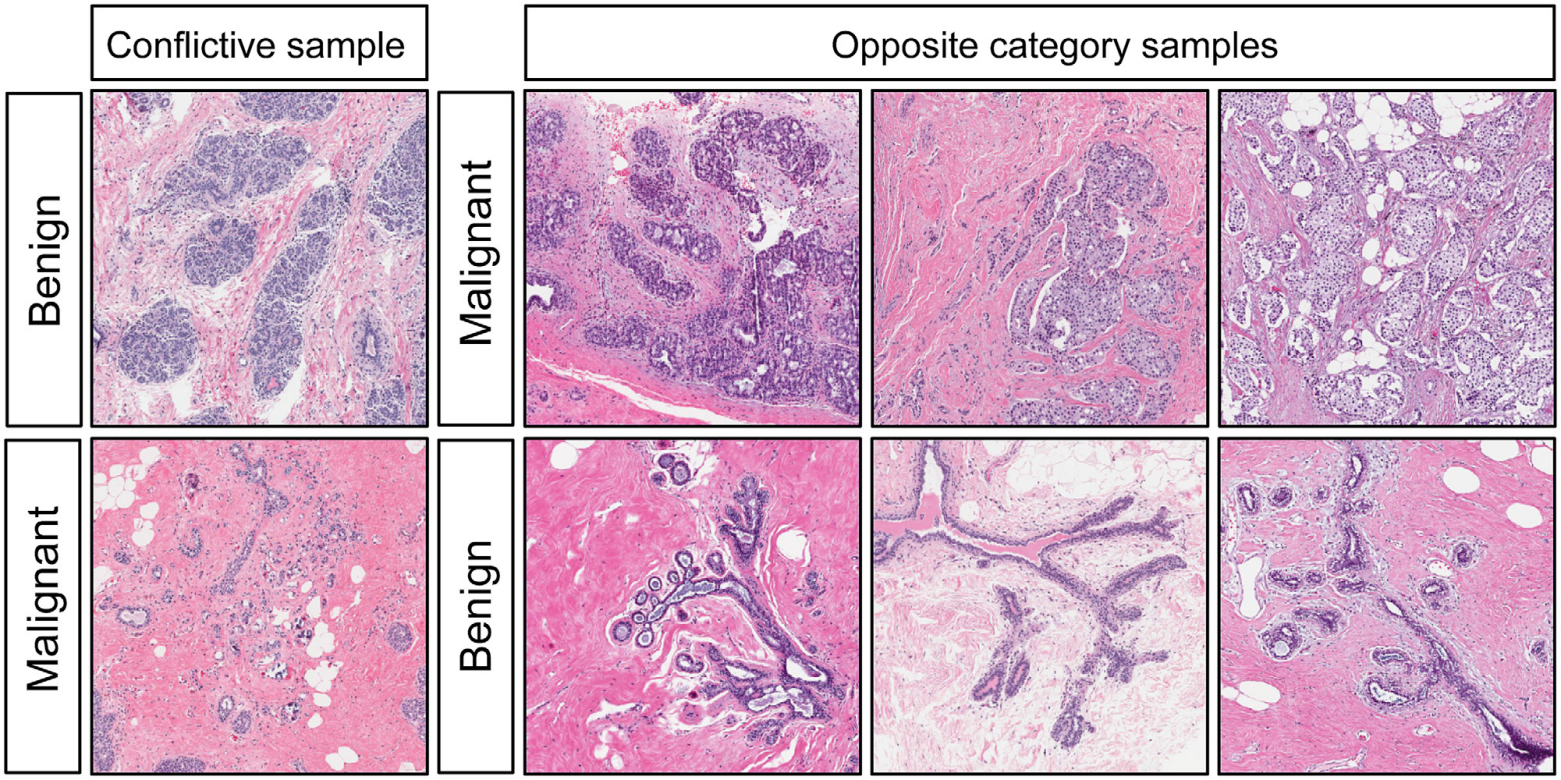
Conflictive histology exemplars. During Experiment 1, some exemplars from a given category looked like exemplars from the other category causing the birds to incorrectly categorize them.

The birds did consistently misidentify a few images, and we wondered if there was a pattern to the errors. Because cancer cells arise from benign (probably stem-cell-like) precursors, the resulting growth patterns and cellular morphologies may deviate only minimally from those of corresponding normal tissues, as reflected in the term, “well-differentiated carcinoma.” Upon review, it appeared that the troublesome images displayed similarities to some of the images in the alternate class. For example, the benign image posing the greatest difficulty (Fig 13, upper left panel) contained breast lobular structures that were indeed benign, but nevertheless highly cellular and densely packed; consequently, at low magnification, they could resemble sheets of cancer cells. In turn, one of the problematic malignant images (Fig 13, lower left panel) proved to be relatively *hypo*cellular, with duct-like structures reminiscent of some of the benign exemplars (Fig 13, bottom). Adding to the potential for confusion, this malignant image also exhibited an abundance of dense eosinophilic (pink) stroma more typical of the normal examples in our training set and unlike most of the other cancer specimens shown to the birds. Thus, the pigeons’ errors did not appear to be random; instead, the least-accurately classified images contained features that were relatively unrepresentative of the rest of the corresponding benign or malignant set, emphasizing the importance of ensuring that images used for training fairly represent the gamut likely to be encountered in future specimens. This, of course, is true for pathology residents’ training as well.

### Effects of image properties on learning speed and accuracy

#### Color and brightness

As noted above, we created monochrome datasets to ensure that the birds were not focusing on trivial differences between benign and malignant histopathology. However, it is also possible to ask whether color information, when present, contributed to classification success, or in other words, whether the birds could be used to assess the impact of image quality variables by measuring the effects of such variables on learning trajectory and final accuracy scores. Color fidelity, for example, is a matter of great interest and concern when digital pathology applications are considered [29].

When a cohort of 4 pathology-naïve pigeons was given monochrome, intensity-normalized images, we found that the birds again learned during training to discriminate them to the same 85% level of accuracy that the prior cohort had achieved on full-color training images. However, their ability to generalize when shown monochrome testing sets was lower, averaging 70% accuracy (Fig 8B) instead of the 85% achieved previously with the full-color testing set (Fig 7). We conclude that color and brightness convey useful, but inessential information, an observation consistent with the fact that until recently, figures in pathology journals and textbooks were frequently presented only in grayscale.

#### Image compression

Lossy compression algorithms—often used for managing otherwise huge image files such as whole-slide scans—decrease storage requirements more effectively than lossless approaches, but the resulting image artifacts can compromise the utility of compressed images for diagnostic purposes [30]. We tested pigeons previously exposed to monochrome images to assess the birds’ ability to discriminate benign from malignant histology when the images were subsequently compressed using medium (15:1) or high (27:1) JPEG compression settings (Fig 3, center and bottom rows). Medium- and high-compression levels, as one might expect, adversely affected pigeons’ performance under *nondifferential* reinforcement (Fig 10, gray bars); however, when *differential* reinforcement was introduced to the training regimen, all compression levels yielded extremely accurate training performance (Fig 10, white bars), indicating the birds’ ability to adapt to changing image properties. In other words, when the pigeons were differentially reinforced for correct responses using the highly compressed images, they were readily able to adapt quite quickly. Humans can also learn to ignore compression artifacts [30] and become accustomed to less-than-perfect visual images. Such an adaptive adjustment, demonstrable with pigeons, might be hard to capture with standard computerized “ideal observer” approaches.

### Mammograms

We extended our exploration of pigeons’ medical-image-relevant capabilities by considering two problems in breast radiology: one, a microcalcification *detection* task; and the second, a mass *discrimination* task, both central to patient management. The birds did well on the first challenge (Fig 11, but poorly on the second (Fig 12). Sensitivity to the presence of microcalcifications was learned and then applied to novel images with much the same success and trajectory as with the histopathology images, with the birds learning within a week, and generalizing reasonably well. The problem of finding little white specks in a complex background would seem to mirror the problem encountered in the pigeons’ native environment, of locating and ingesting seeds distributed in a visually cluttered environment—an obvious survival skill.

In contrast, evaluating the malignant potential of detected breast masses (Fig 5) can be very challenging [18]. A panel of radiologists (tested by EAK) achieved an accuracy rate of about 80% when viewing images of the relatively subtle masses employed in this study. The pigeons also found this task to be difficult, and in fact took many weeks to distinguish the training-set masses, rather than the days needed with the prior histopathology studies (Fig 12A). Unlike the histology and calcification tasks, in which all birds performed similarly, some birds proved to be more skilled during this phase; only two pigeons reached 80% correct classification, and the other two plateaued at 60%. More importantly, none of the birds performed well when they were shown novel testing stimuli, with even the high-performers during the training period doing no better than chance (Fig 12B). These data suggest that the birds *memorized* the masses in the training set, but never learned to key in on those features (for example, stellate margins) that can correlate with malignancy. This result reflects the difficulty of the task, with which even human experts struggle, and indicates that birds may be relatively faithful mimics of the strengths and weaknesses of human capabilities when viewing medical images.

### Human and avian vision systems: implications for potential utility

Pigeons have low-level vision capabilities that generally parallel those of humans. This is not surprising as the visual systems of all amniotes share major organizational properties [31, 32]. The specific underlying mechanisms of visual learning appear to be similar between avians and primates, despite some 300 million years of evolutionary divergence and structural neuroanatomical disparities: feedforward and hierarchical processing seem to dominate [15]. Some species-specific differences may also be significant: while pigeons can indeed distinguish human male from female faces, the primary features used appear to be largely texture-based [16]; by contrast, humans probably have additional recognition tools for faces (and emotions) that are not available to avian observers.

However, these higher-level differences are probably not relevant to the studies described here. So, on balance, it appears that pigeons’ visual discrimination abilities and underlying neural pathways are sufficiently similar to those of humans when challenged by medical image evaluation tasks as to have potential practical significance. Characteristics that are helpful in distinguishing benign from malignant histology, for example, may be apparent in overall tissue architecture variation as well as in differences in cell nuclear size, shape, or other features. Pathologists use such multi-scale features as they examine lesions at magnifications from 1× (sometimes by holding a glass slide against a white coat sleeve to assess “blueness” as an indicator of overall cellularity) to 40×, at which power nuclear chromatin distribution, for example, can be discerned. In fact, the pigeons were able to distinguish benign from malignant histologies at simulated magnifications from 4× to 20×, indicating that they were able to perform accurate classification across about a 20-fold resolution range. This ability is consistent with their status as primarily vision-directed animals, and reflects their facility with locally weighted [33], texture-based discrimination tasks, at which they are at least as good as when they are confronted with object-, geometric-, or feature-based discrimination problems [34].

If pigeons were relying on textural clues, then this observation would parallel many current machine-learning approaches that depend on texture features to achieve strong and generalizable (medical) image segmentation performance [35, 36]. Future studies in which birds are exposed to images in which certain low-level pixel-based features are either emphasized or suppressed could help pinpoint which properties contribute most to detection or classification performance [16, 37, 38]. Such studies might also indicate how best to process and present medical images to human observers for maximal saliency, as well as motivate future machine-learning strategies.

Overall, our results suggest that pigeons can be used as suitable surrogates for human observers in certain medical image perception studies, thus avoiding the need to recruit, pay, and retain clinicians as subjects for relatively mundane tasks. Beyond cost and convenience, there may be other advantages to using pigeons. For example, the level of observer expertise for any given study can be set by design, from naïve to expert—a distribution that may hard to achieve with readily available human viewers. In addition, pigeons can be used repeatedly in studies designed to explore a wide set of parameters [33] and more cases could be considered than human observers would ever tolerate, thereby yielding greater statistical power. Pigeon-based experiments could also enable more rigorous analysis of the effects of lesion (target) prevalence on detection [39]. Such studies involving *adaptive* interaction between observers and the observed are hard to replicate using only mathematical ideal observers.

In addition to content-based studies, it may also be helpful to use pigeons to explore the impact of technical aspects of color fidelity, display parameters (e.g., contrast, brightness, gamma), and image compression artifacts on observer performance, studies that can present logistical challenges that are associated with assembling appropriate and affordable expert human panels [40]. For example, color fidelity in digital pathology is a major issue for image capture and presentation [41]. As an example of how pigeons could be used in this context, we found that complete removal of color information had measurable effects on pigeon classification accuracy and learning trajectory, but the birds were nevertheless able to overcome these performance deficits with increased exposure to the monochrome images, aspects of discriminative performance that may faithfully reflect human behavior as well.

Of course, pigeons are not the only animals to have shown abilities relevant to medical diagnoses. Other animals with special perceptual skills have been proposed as front-line diagnosticians, for example, dogs sniffing out prostate or ovarian cancer [42, 43] or giant African pouched rats detecting tuberculosis [44]. We are not (yet) proposing such a role for pigeons. Instead, we have shown that pigeons can be effective, tractable, relevant, informative, statistically interpretable, and cost-effective medical image observers. Their performance on visual discrimination tasks might prove useful in guiding basic vision research into feature saliency and could play a key role in the development of computer-assisted medical image recognition tools.

## Supporting Information

S1 File Pathology image database. (PDF)

S2 File Radiology image database. (PDF)

S3 File Experimental data. (ZIP)
