# Supplementary material for: Pigeons (Columba livia) as Trainable Observers of Pathology and Radiology Breast Cancer Images
Source: PLoS One. 2015 Nov 18;10(11):e0141357. doi: 10.1371/journal.pone.0141357 (PMC4651348; doi:10.1371/journal.pone.0141357)

4x Normal Set A

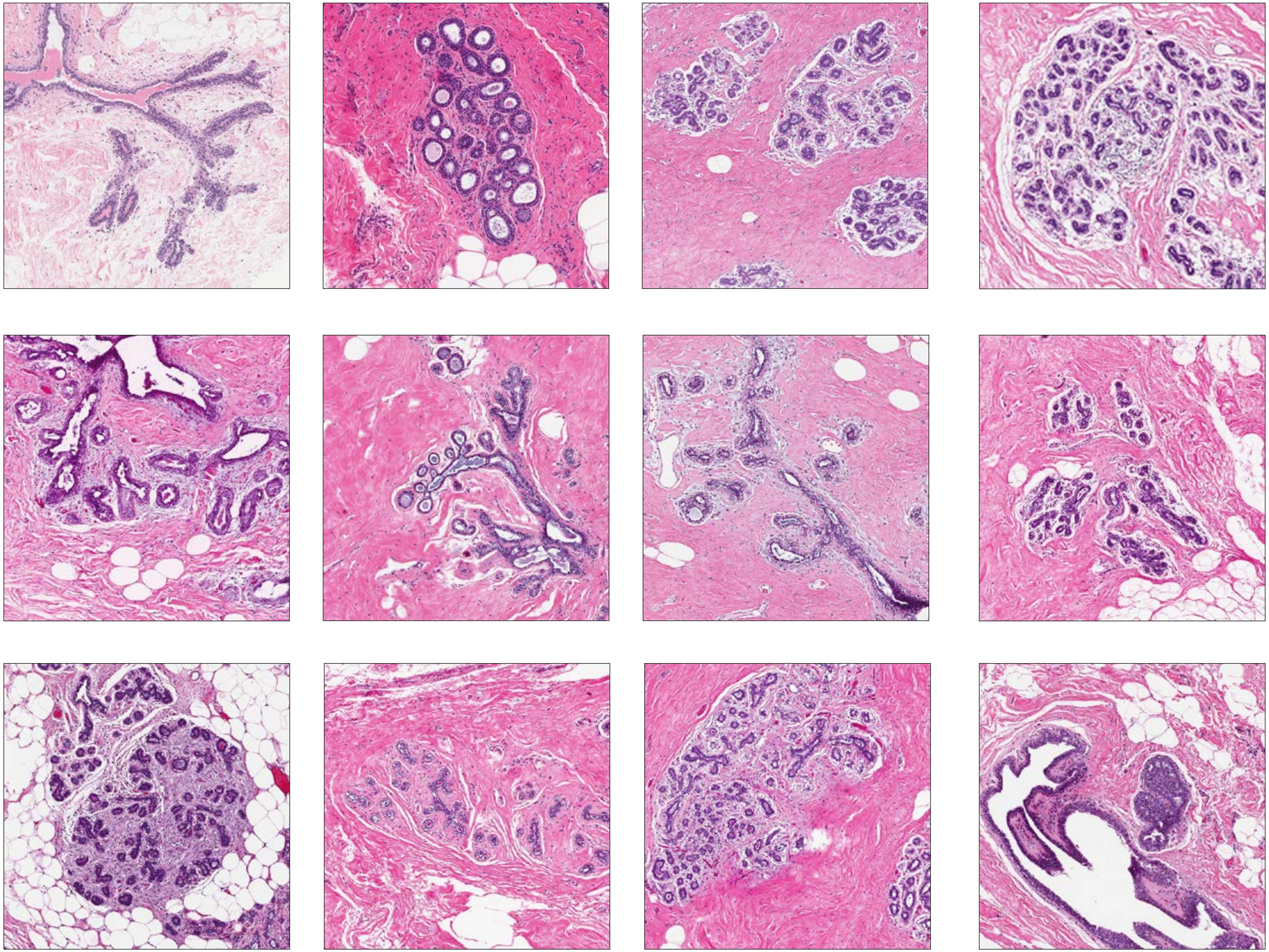

4x Normal Set B

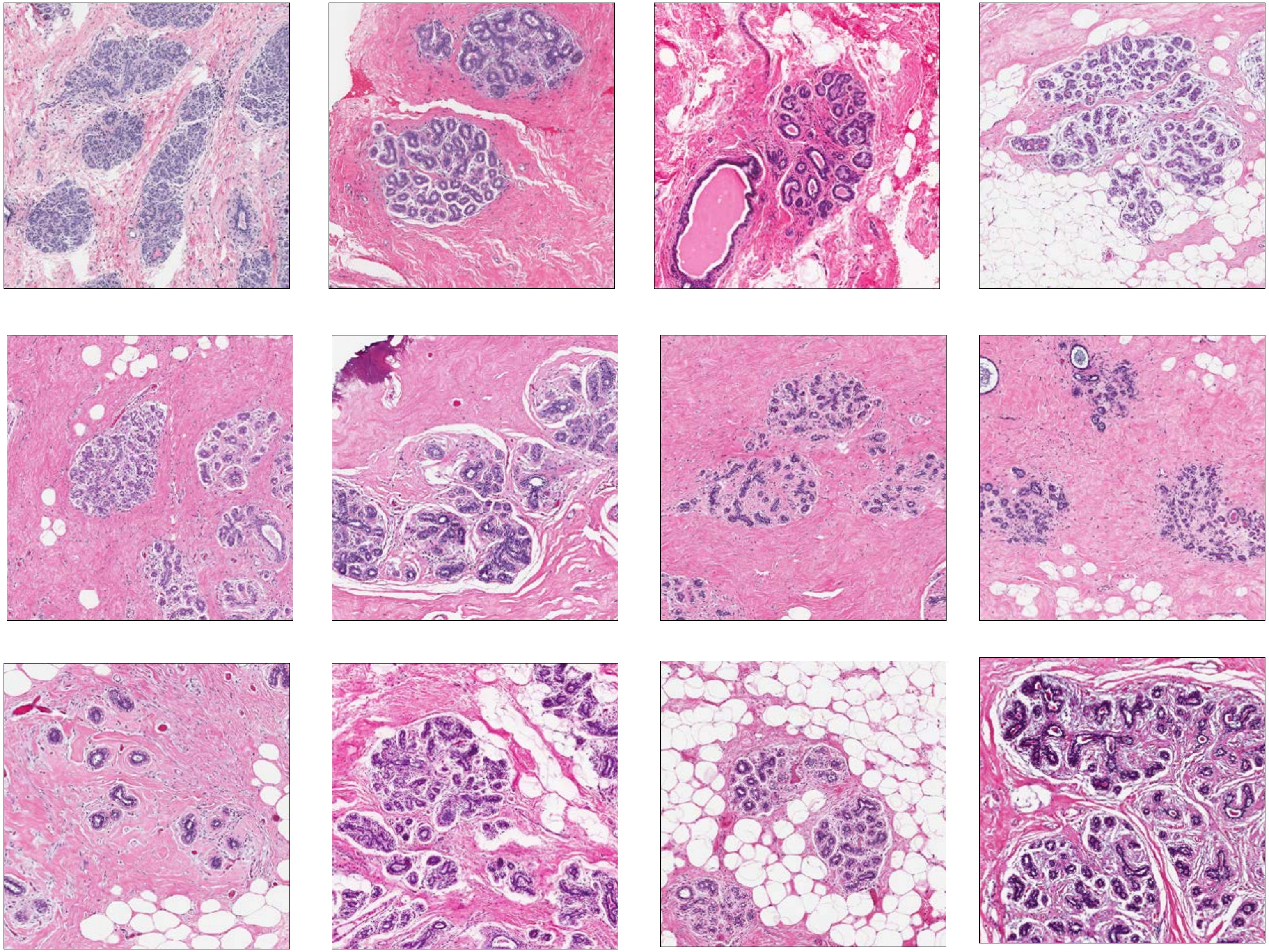

4x CA Set A

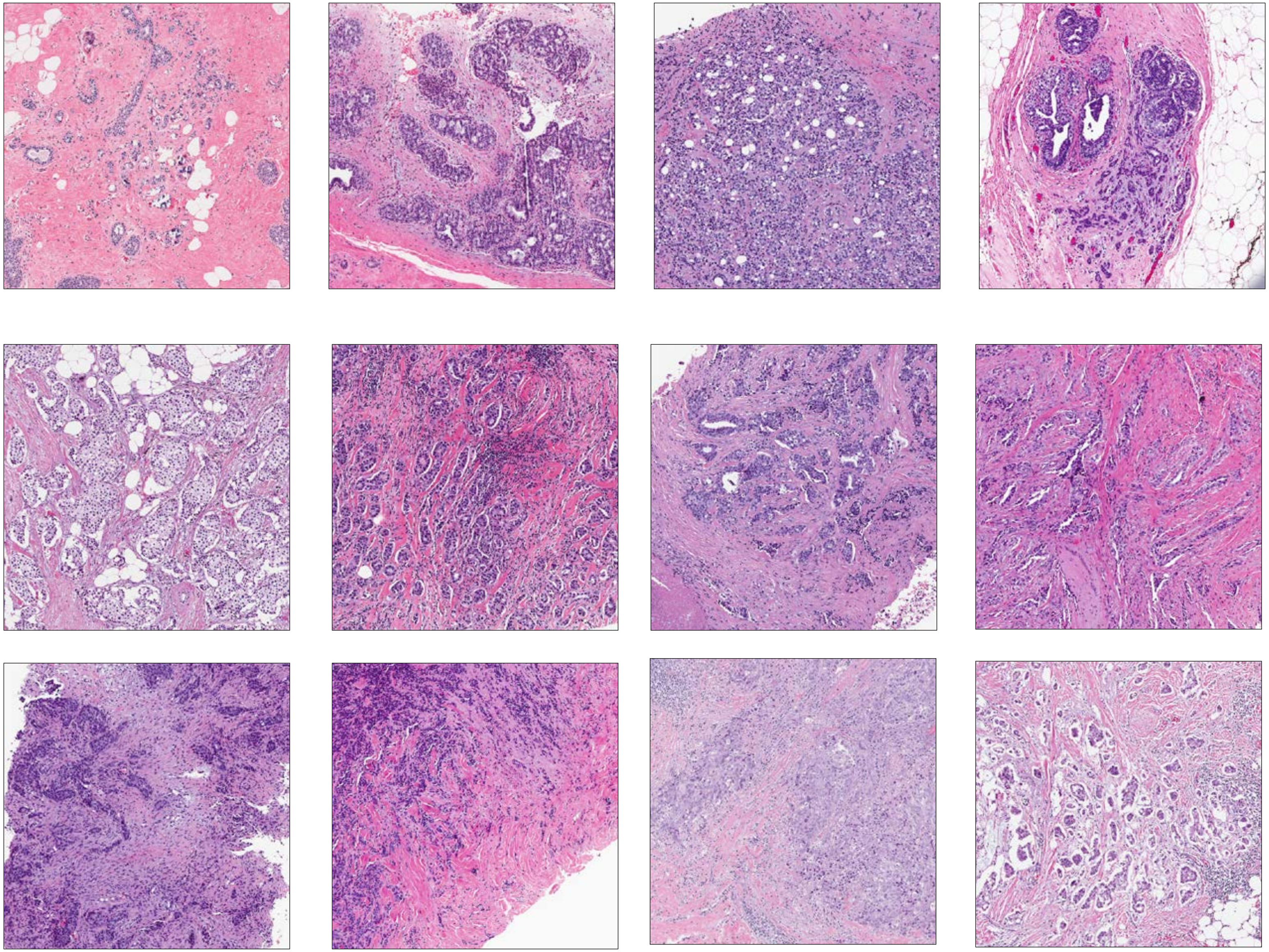

4x CA Set B

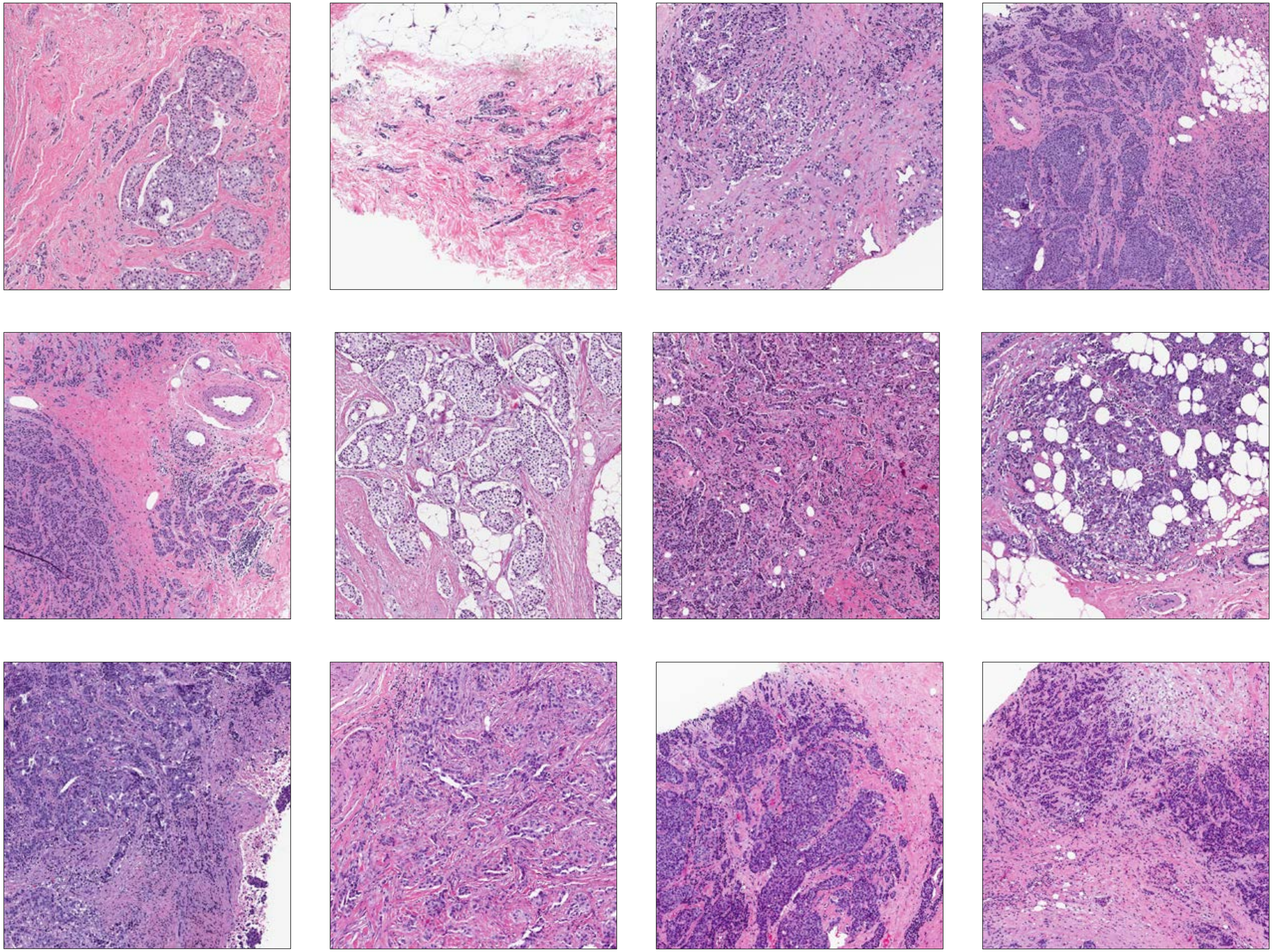

10x Normal Set A

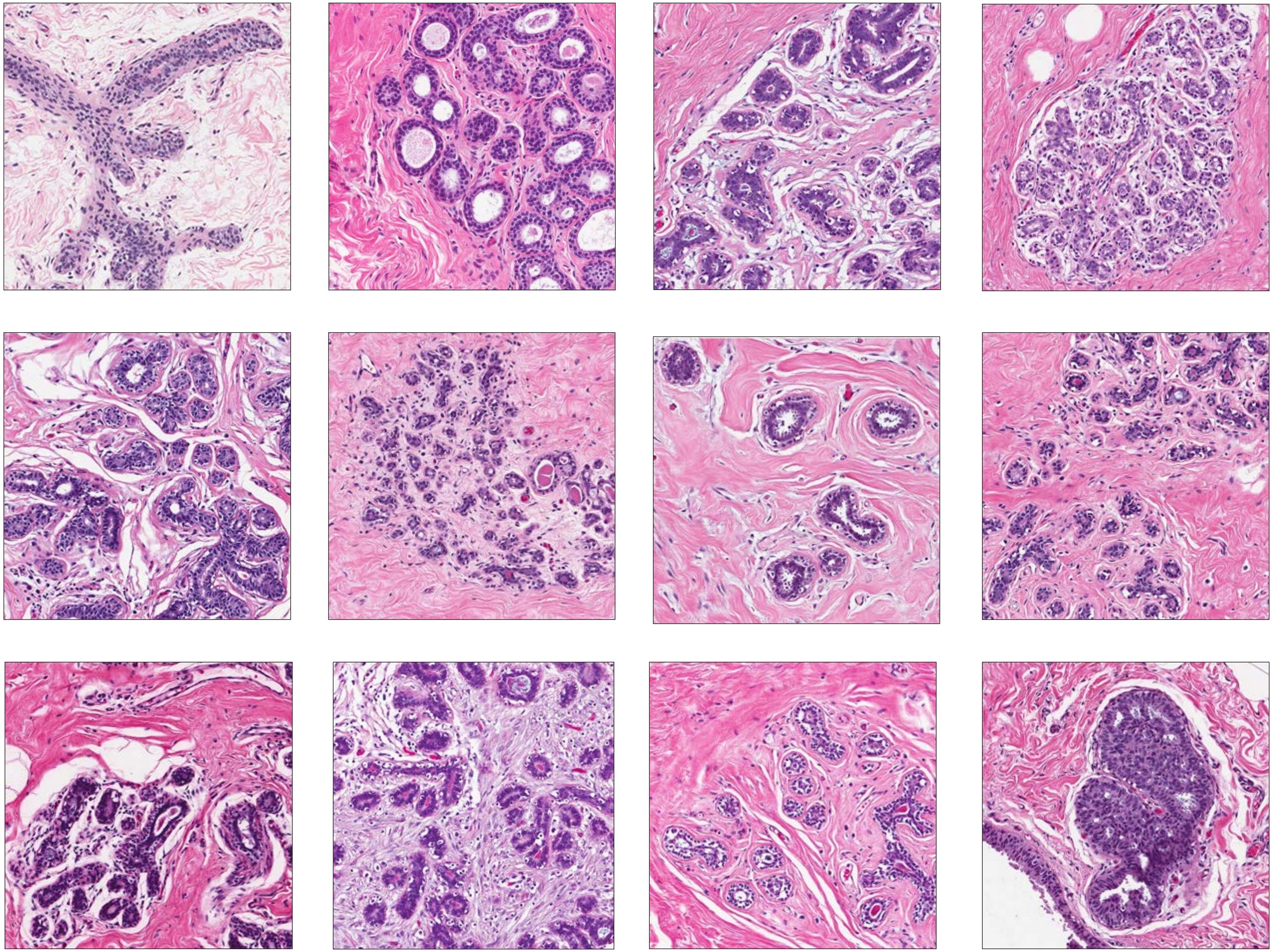

10x Normal Set B

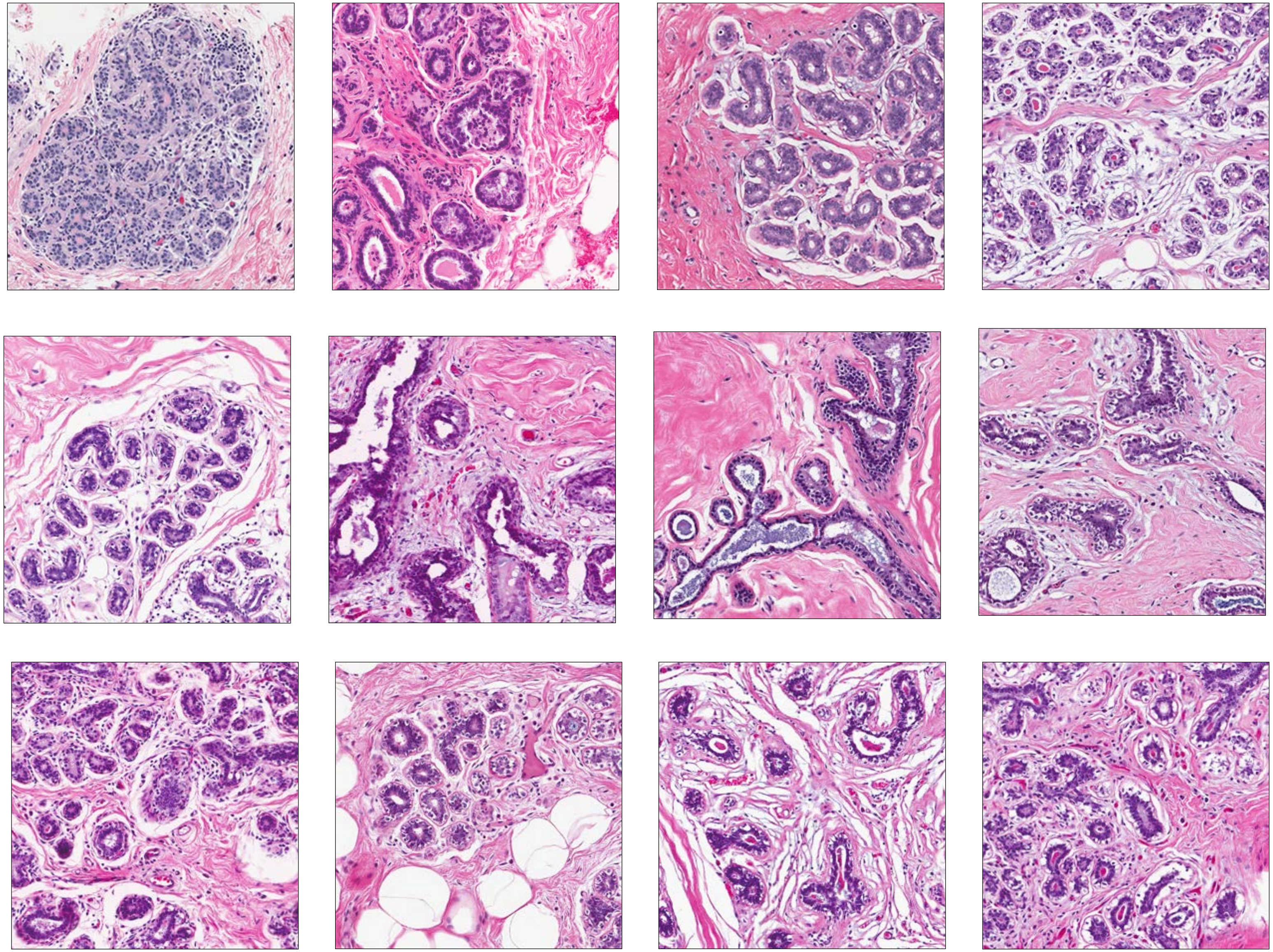

10x CA Set A

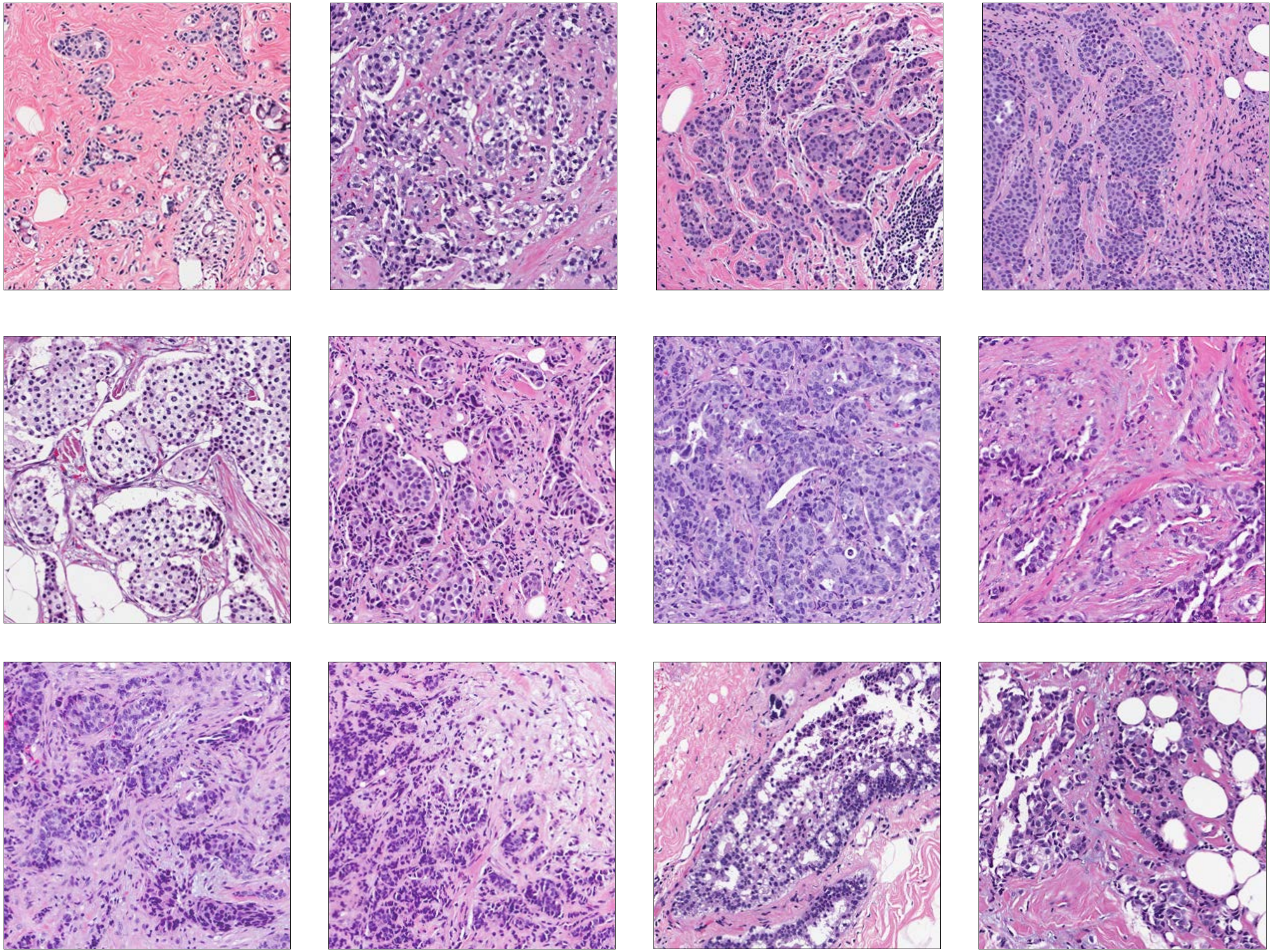

10x CA Set B

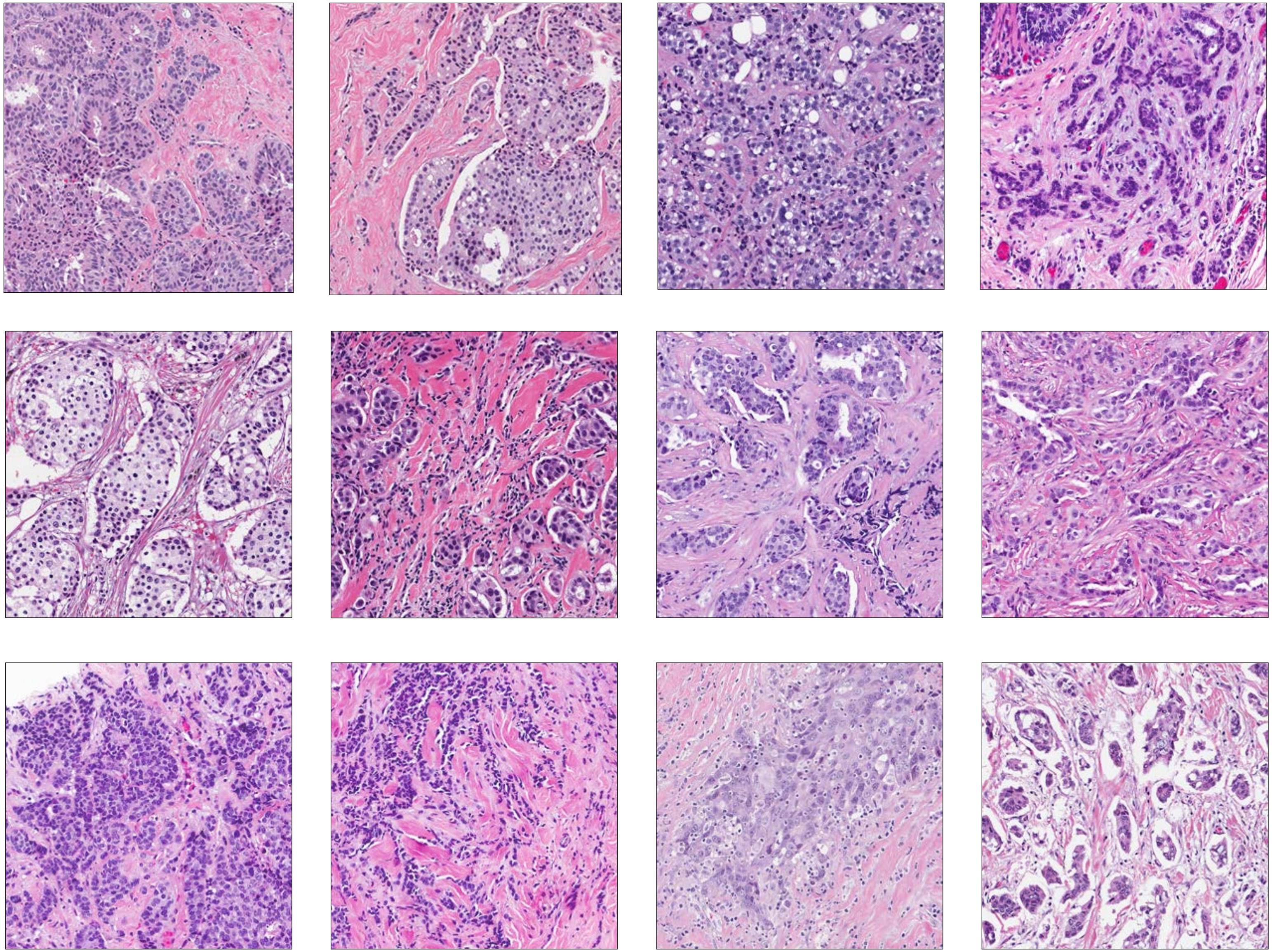

20x Normal Set A

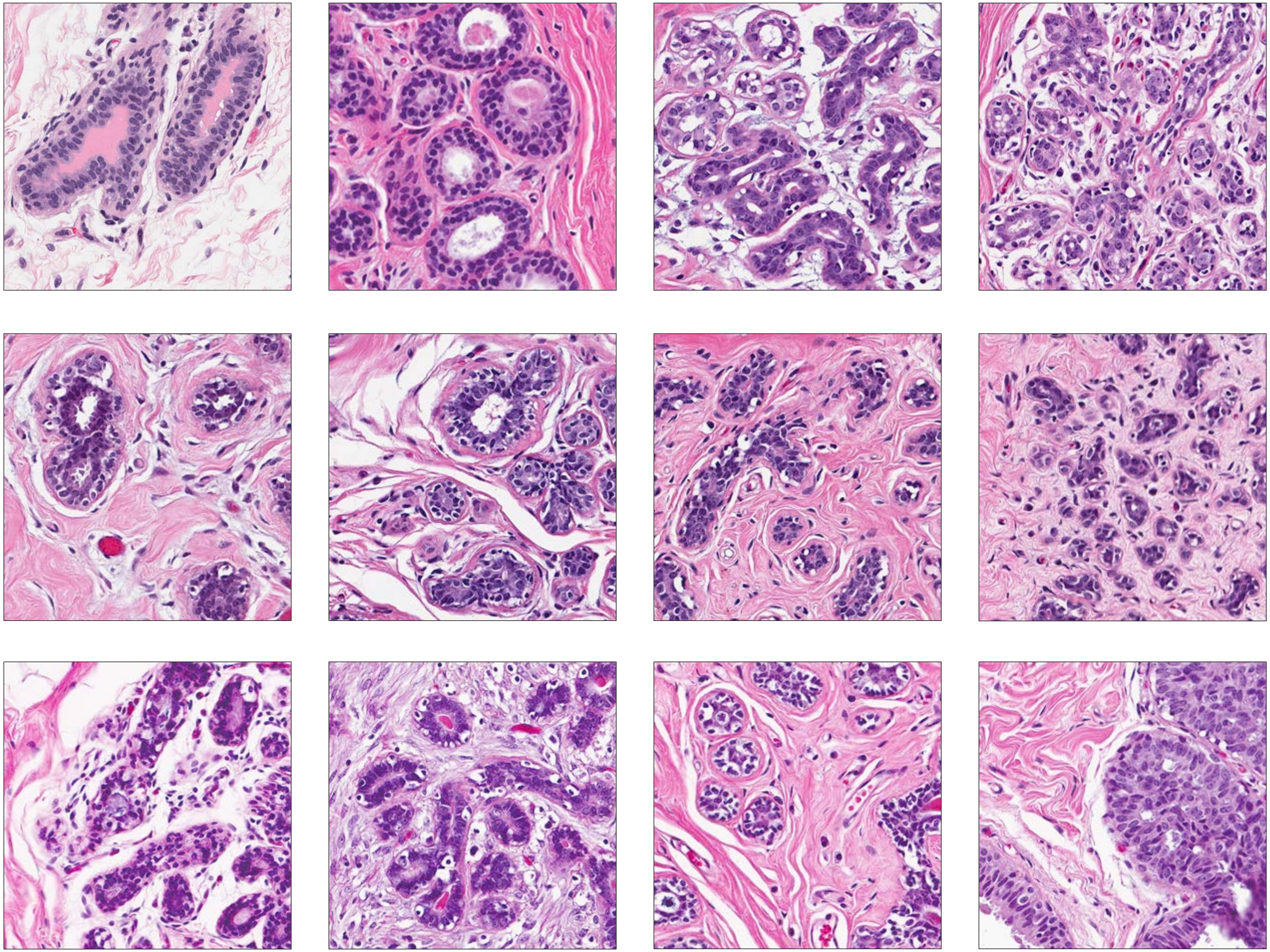

20x Normal Set B

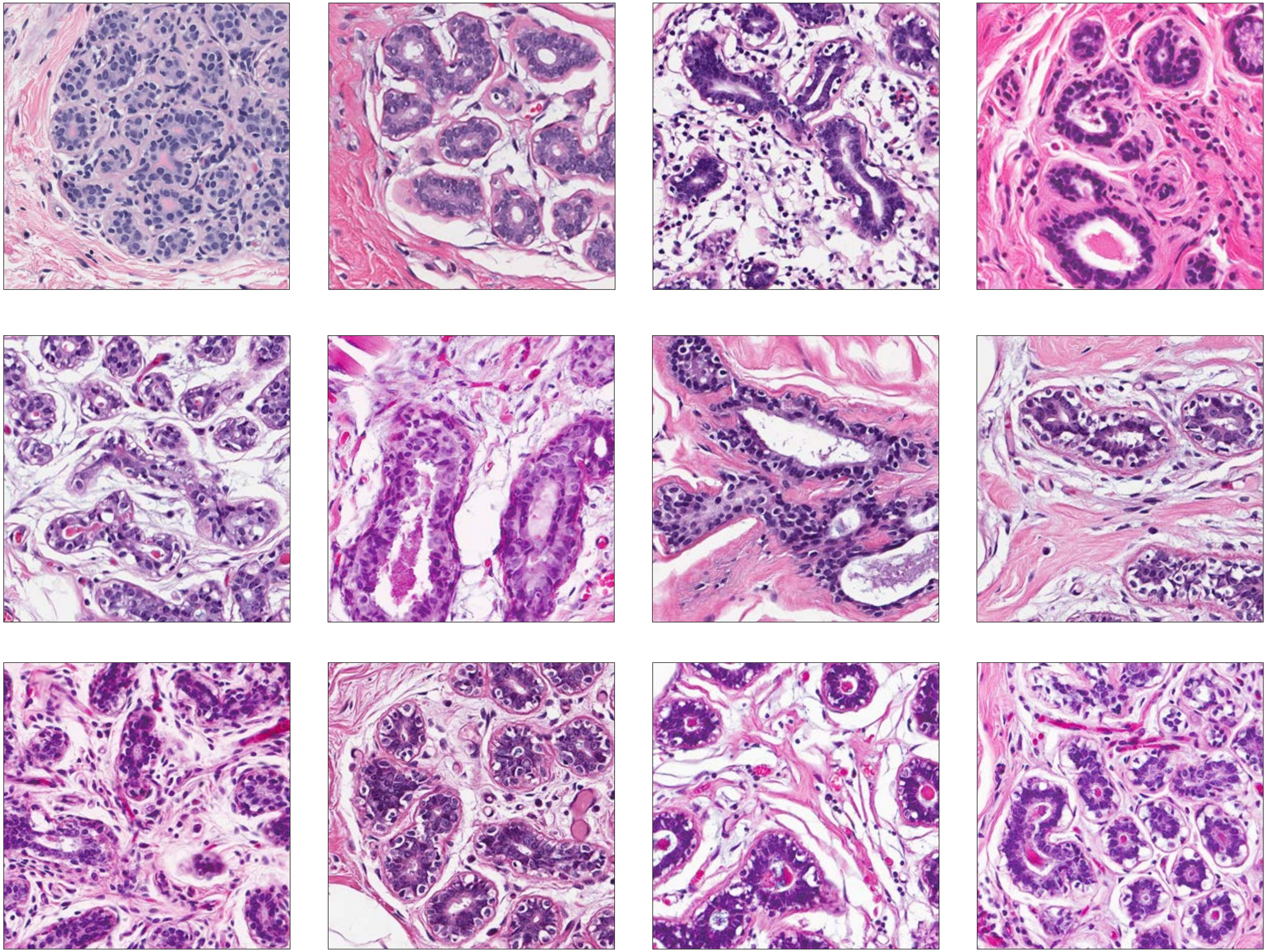

20x CA Set A

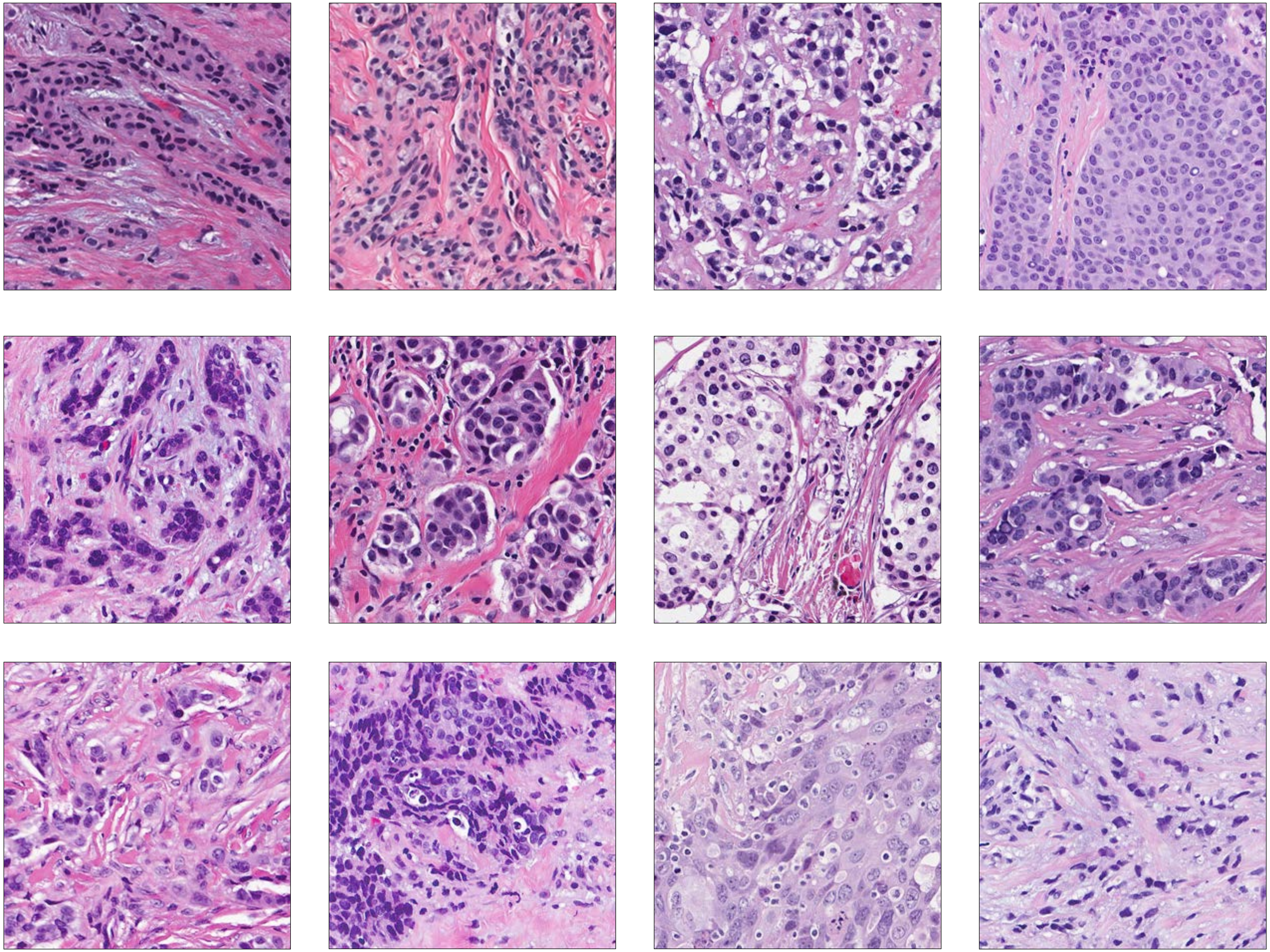

20x CA Set B

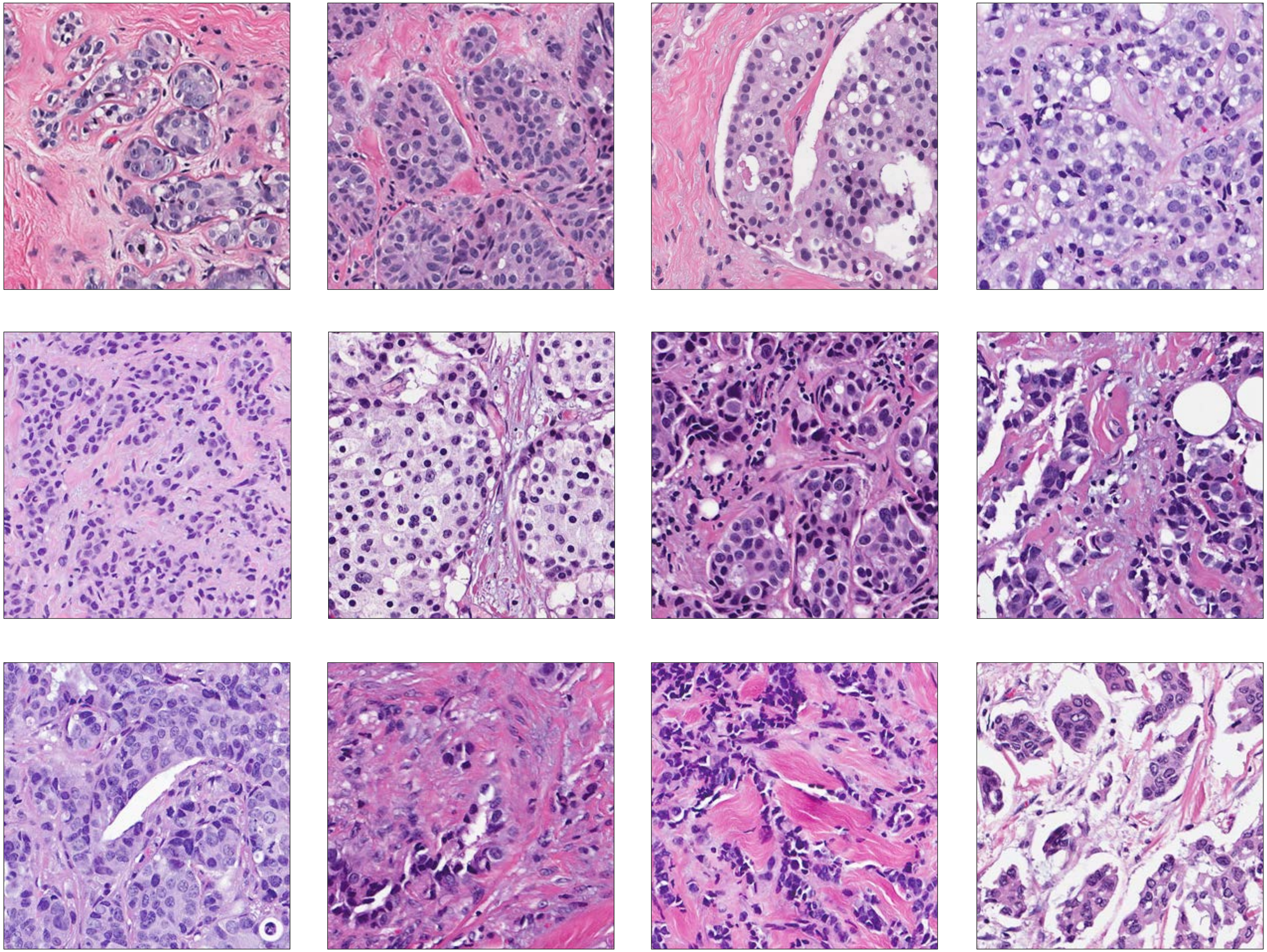

10x Normal Monochrome Set A

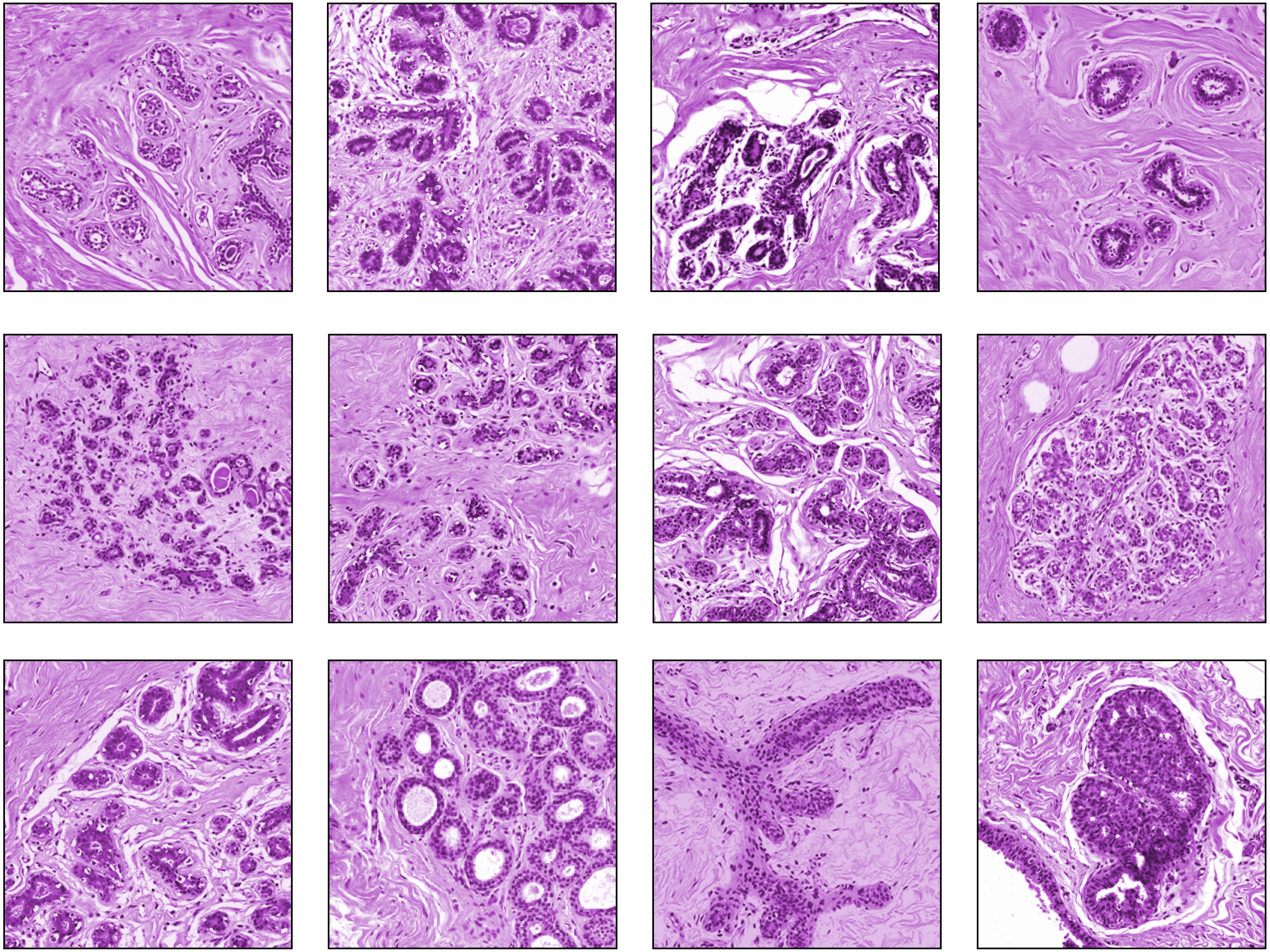

10x Normal Monochrome Set B

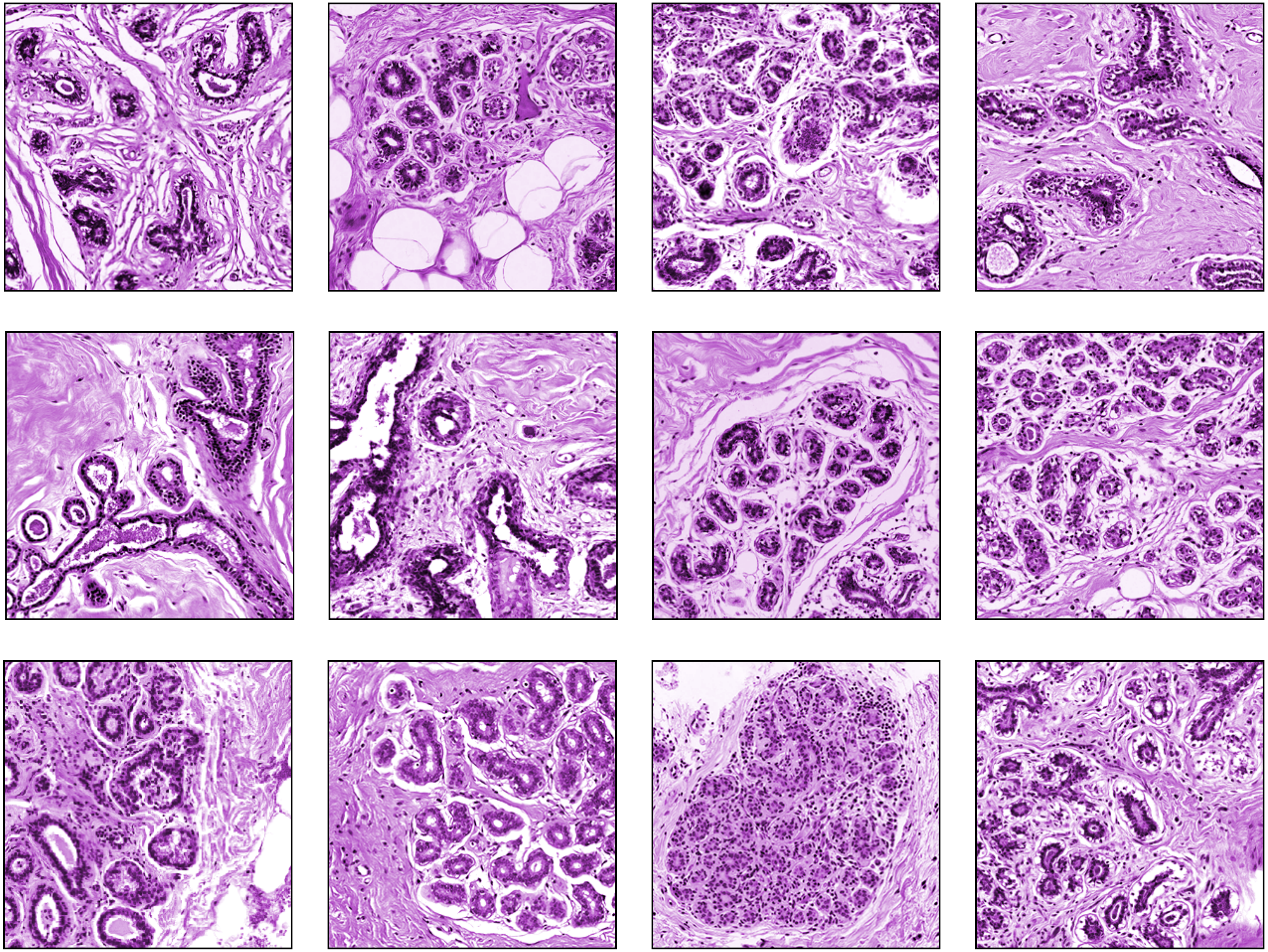

10x Cancer Monochrome Set A

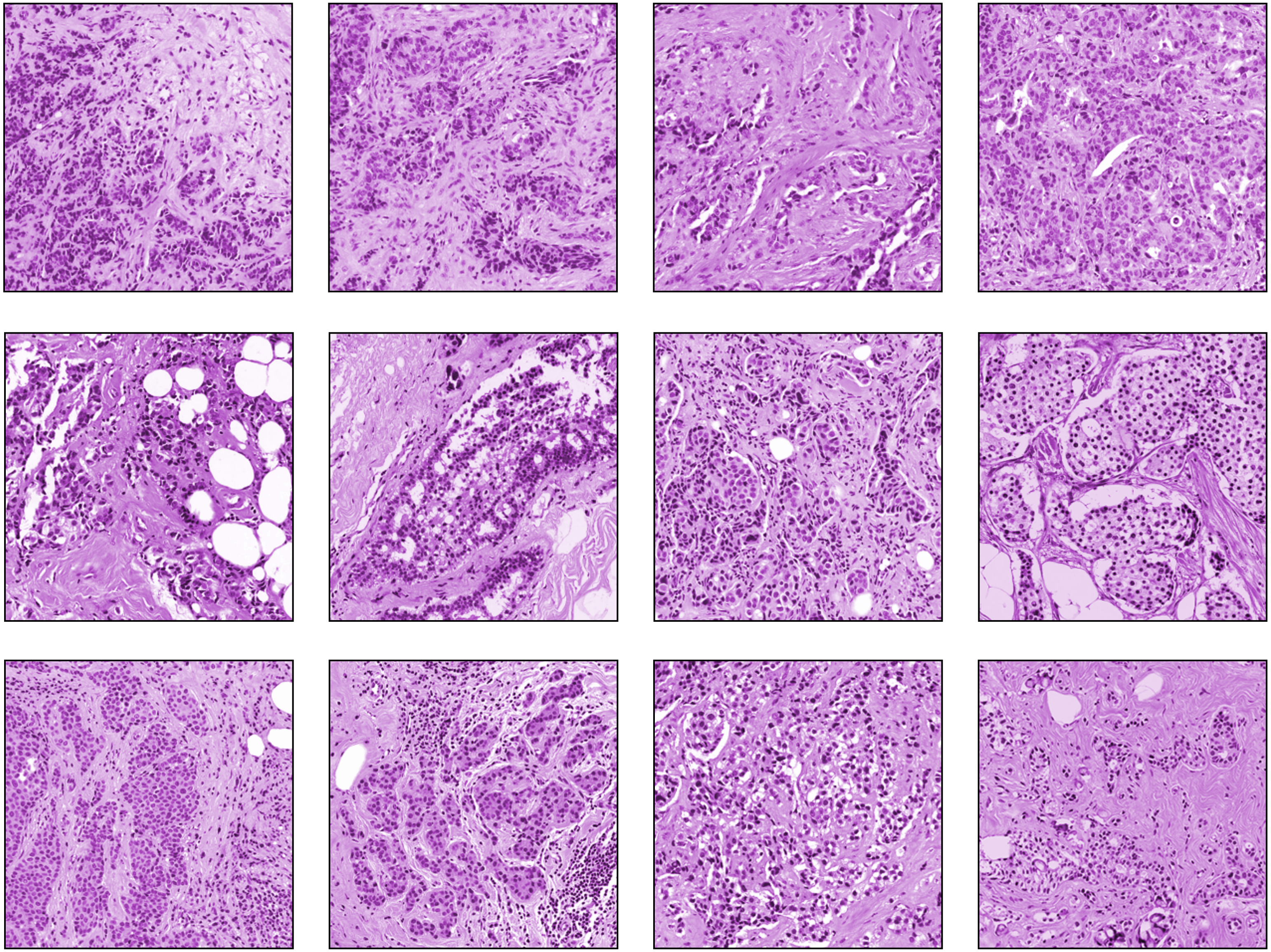

10x Cancer Monochrome Set B

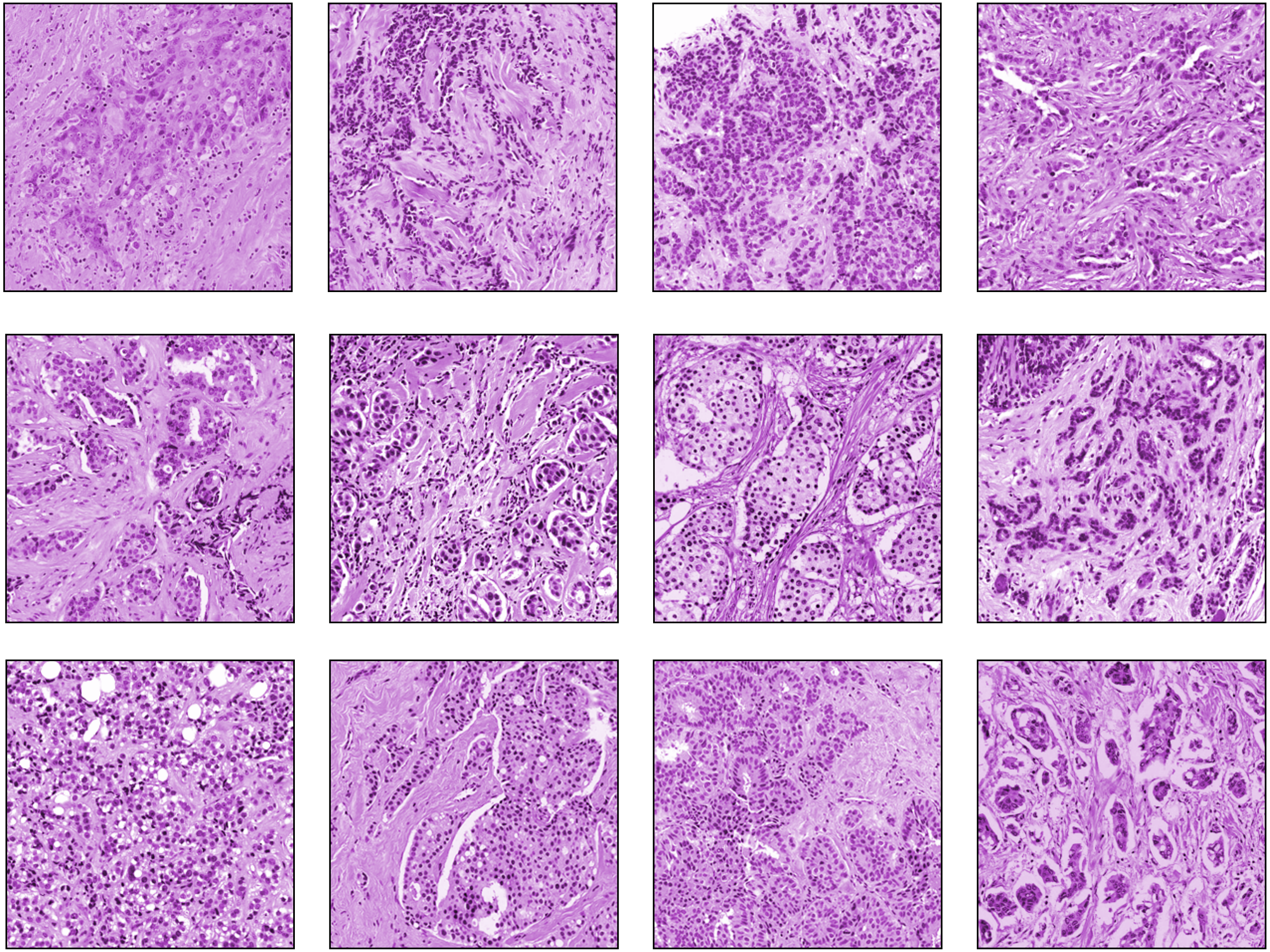

10x Normal Monochrome 1:15 JPEG Set A

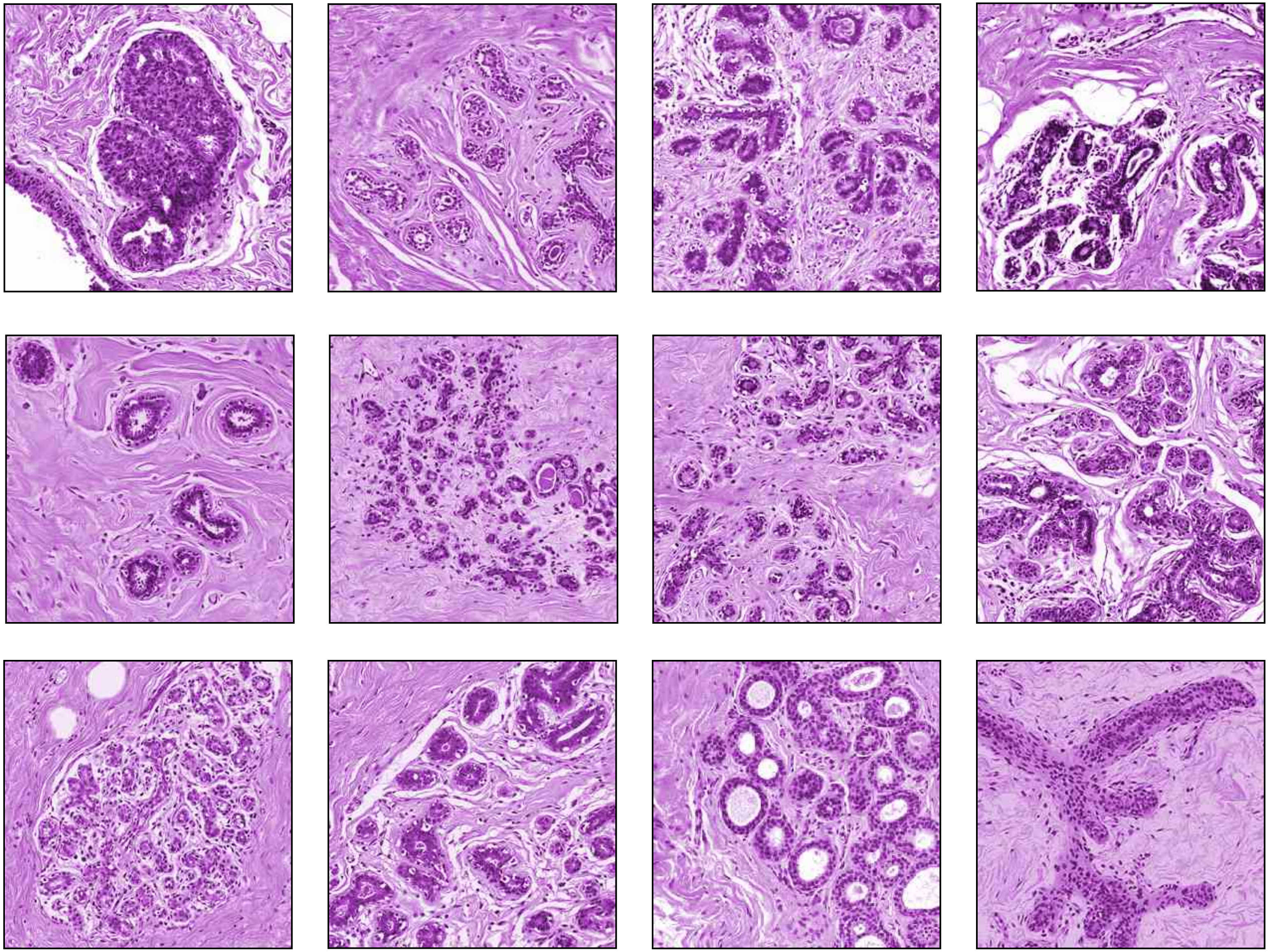

10x Normal Monochrome 1:15 JPEG Set B

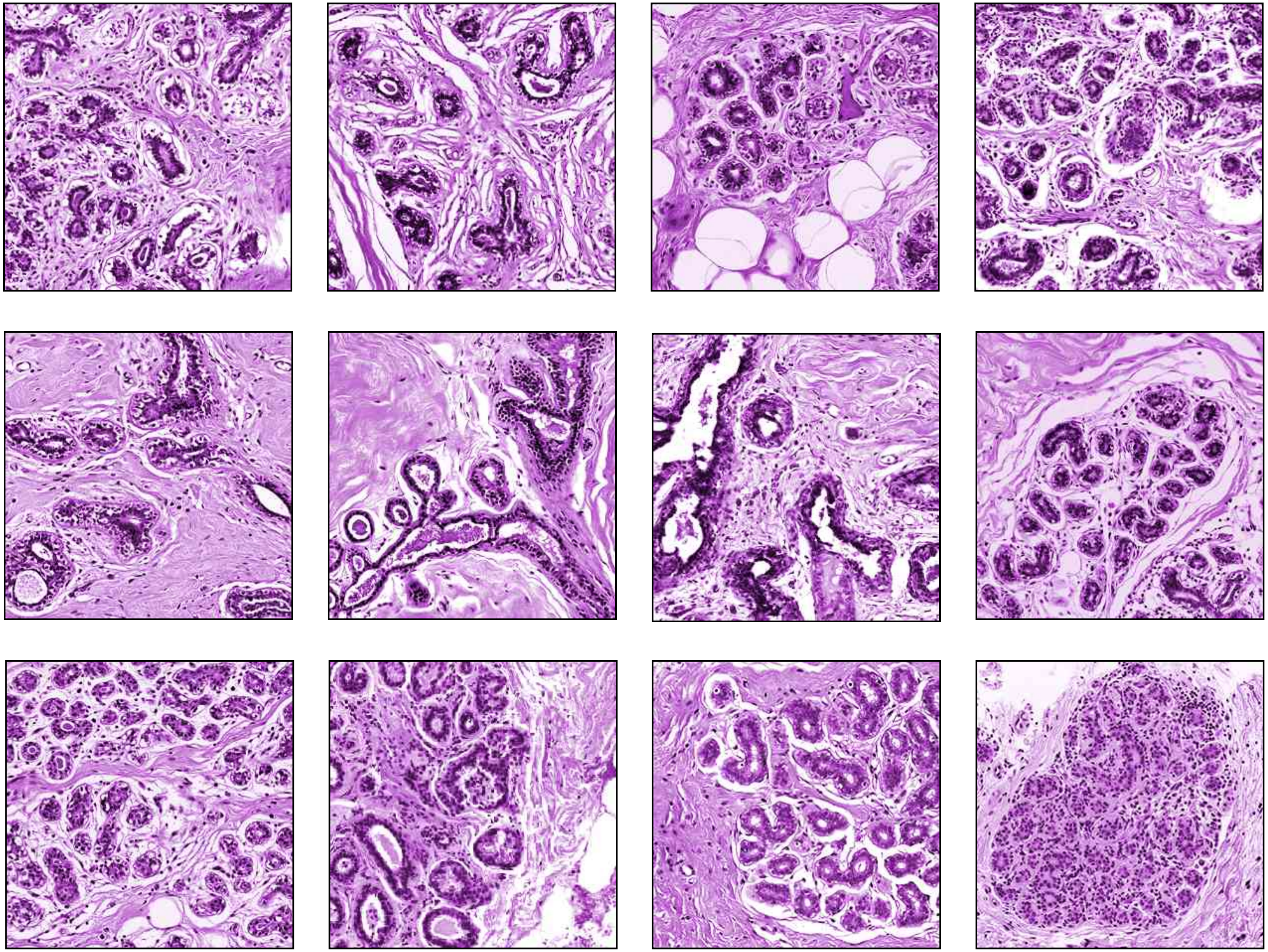

10x Cancer Monochrome 1:15 JPEG Set A

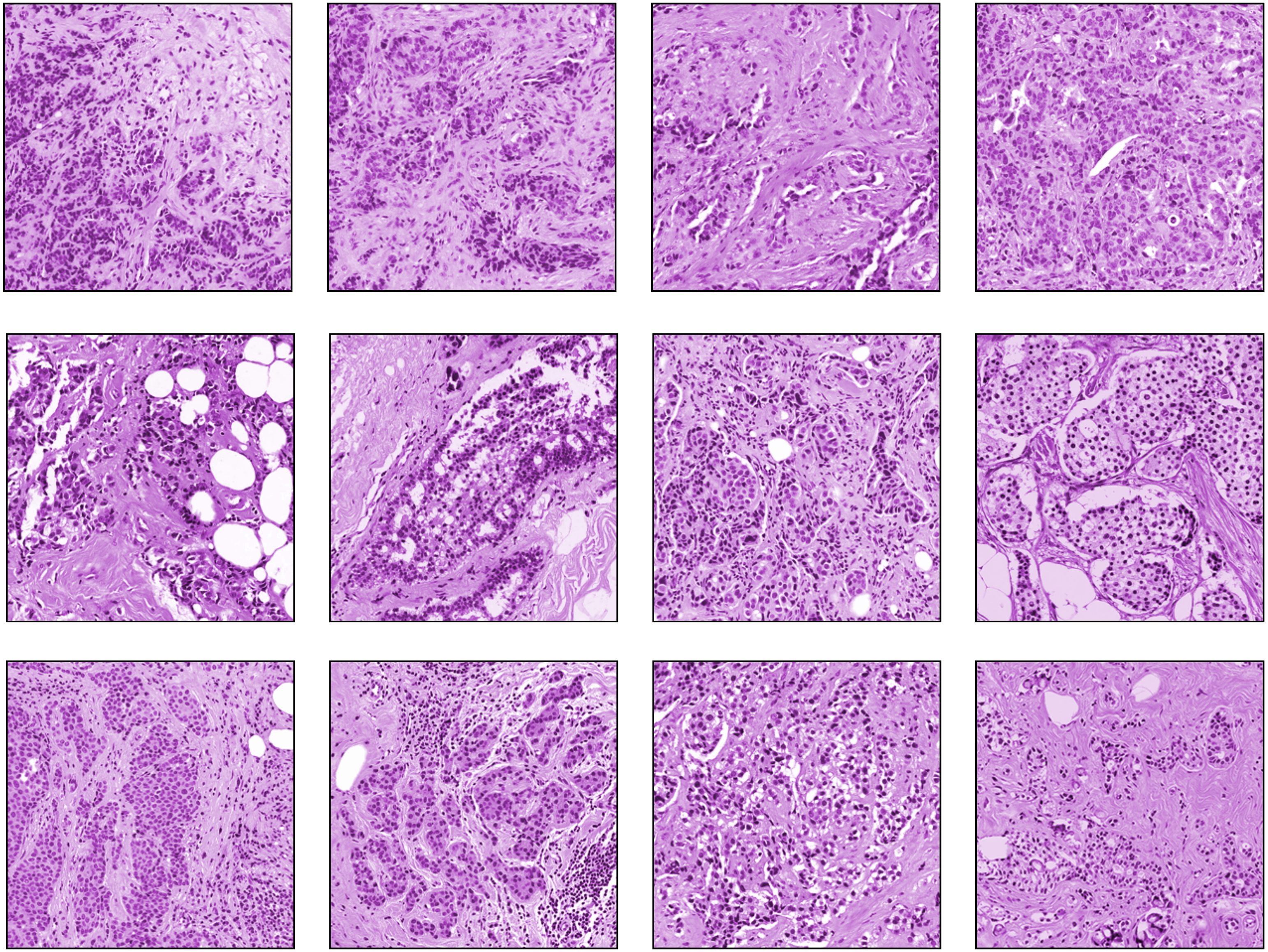

10x Cancer Monochrome 1:15 JPEG Set B

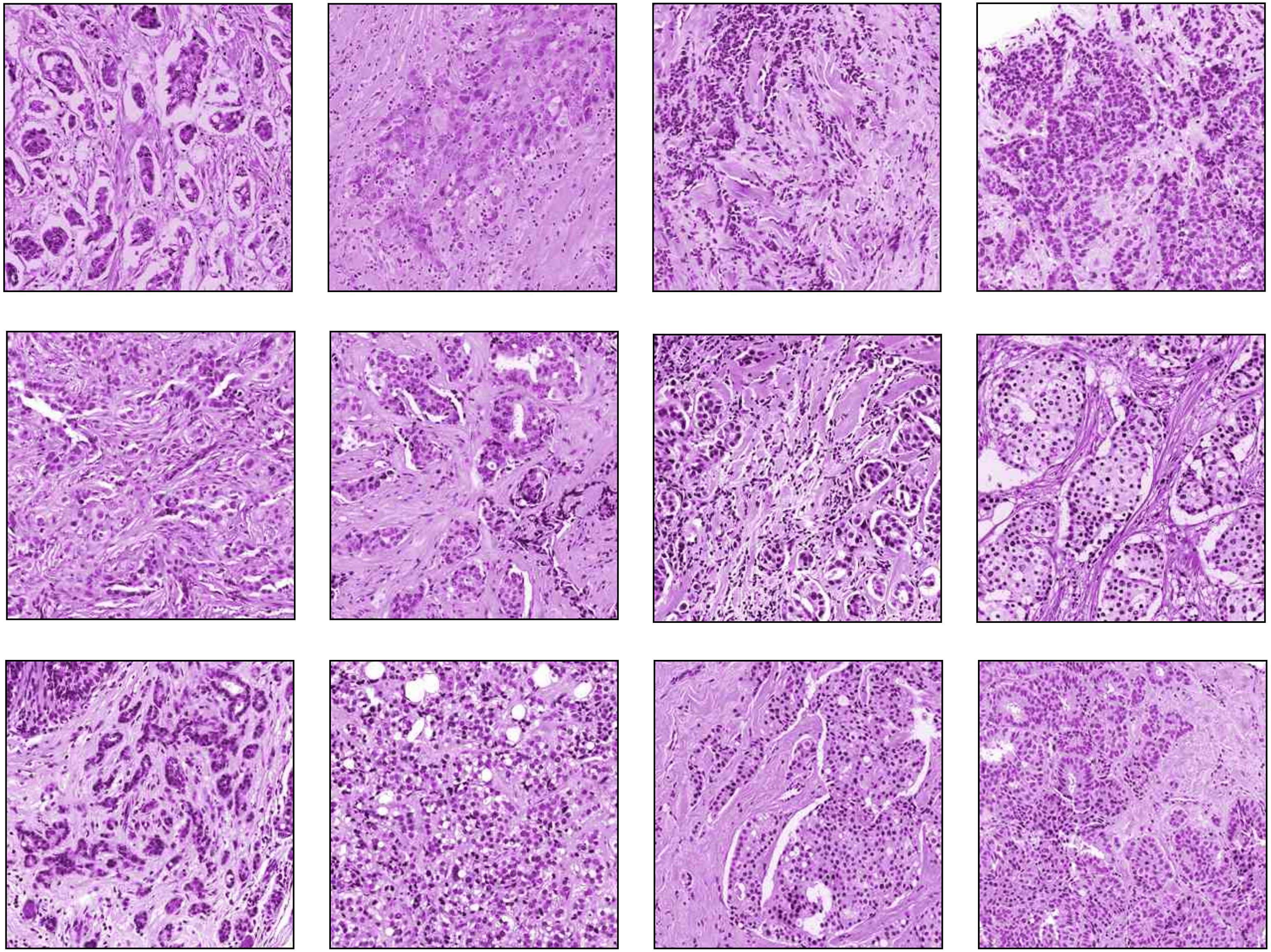

10x Normal Monochrome 1:27 JPEG Set A

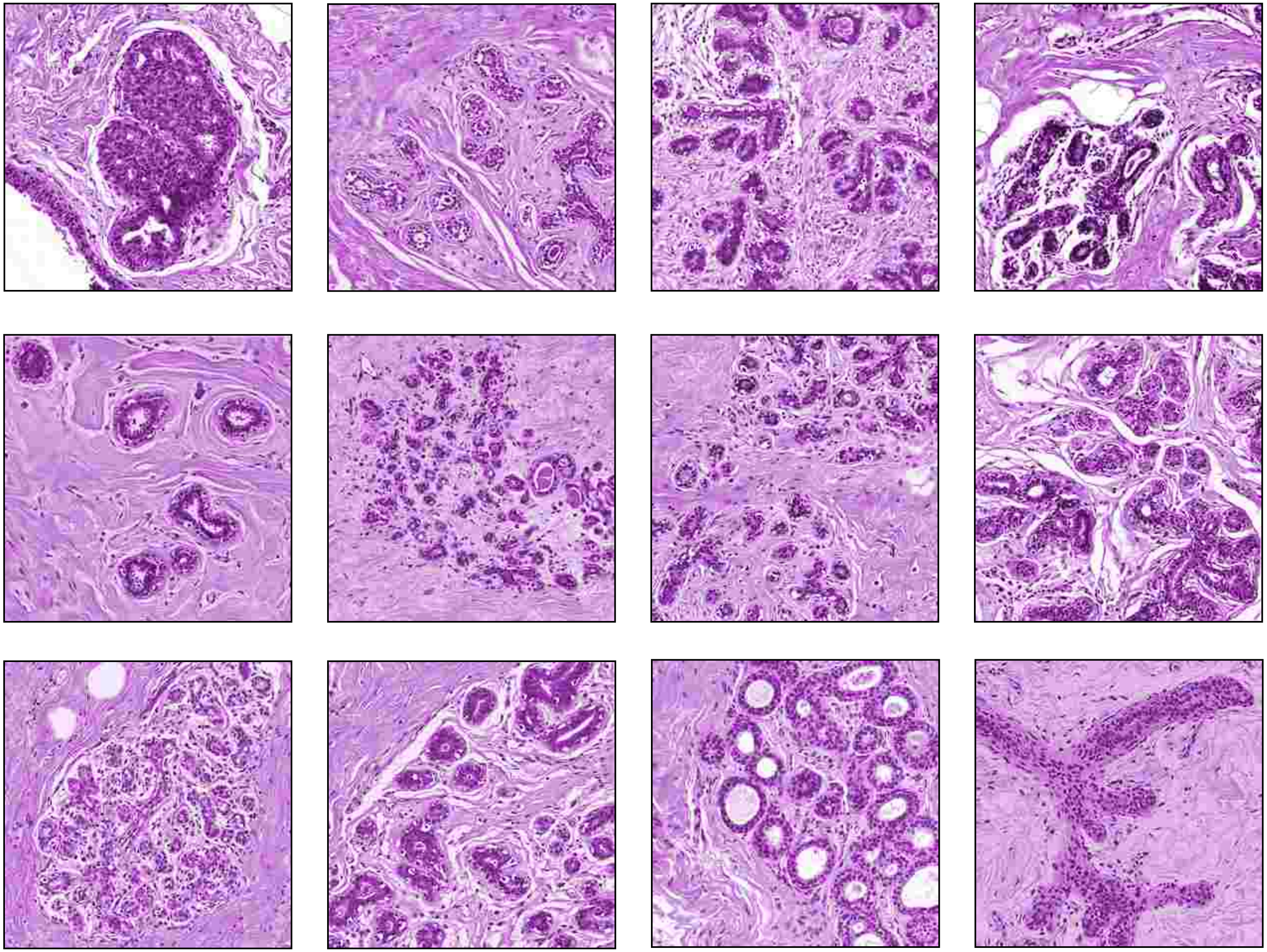

10x Normal Monochrome 1:27 JPEG Set B

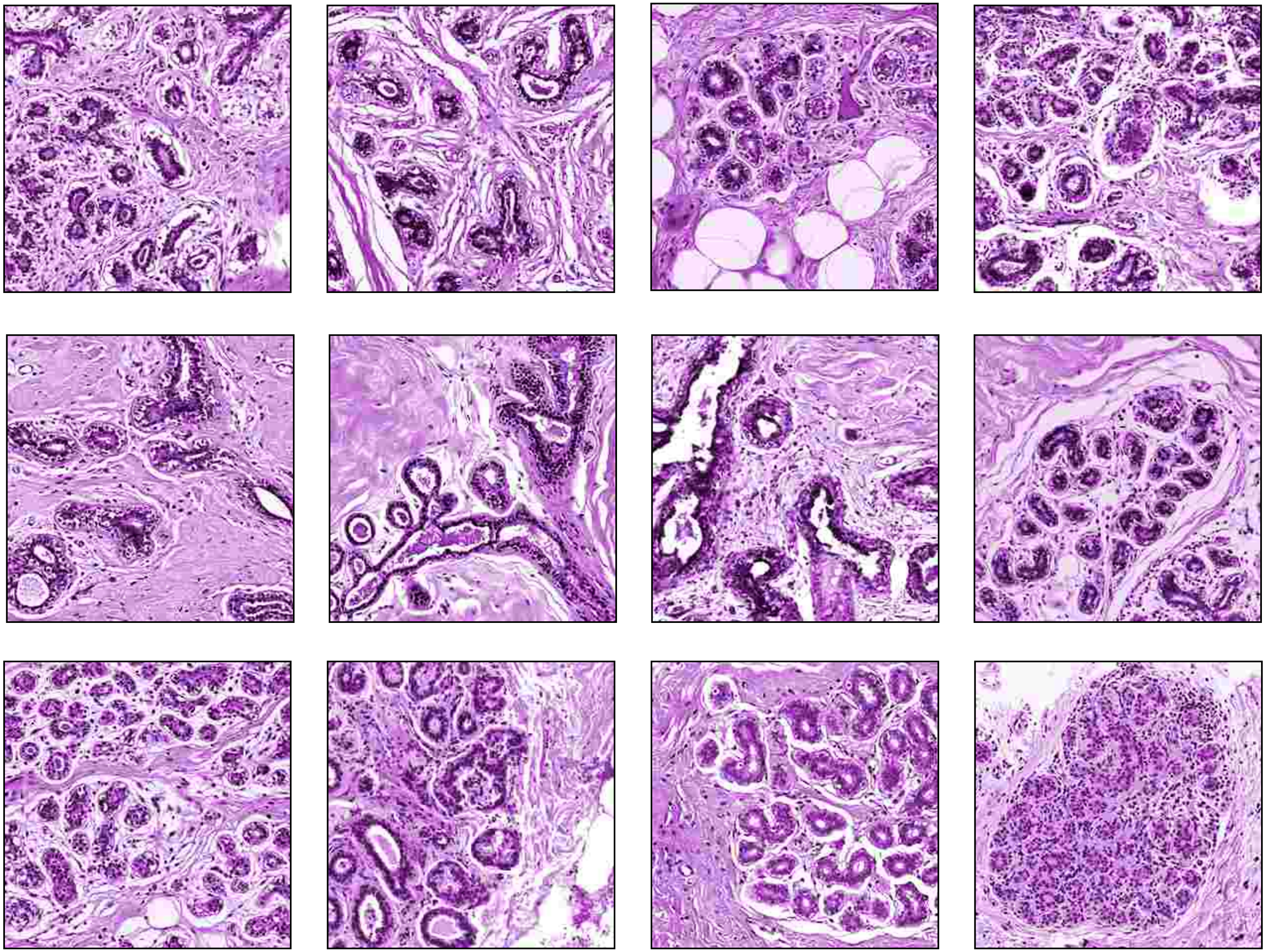

10x Cancer Monochrome 1:27 JPEG Set A

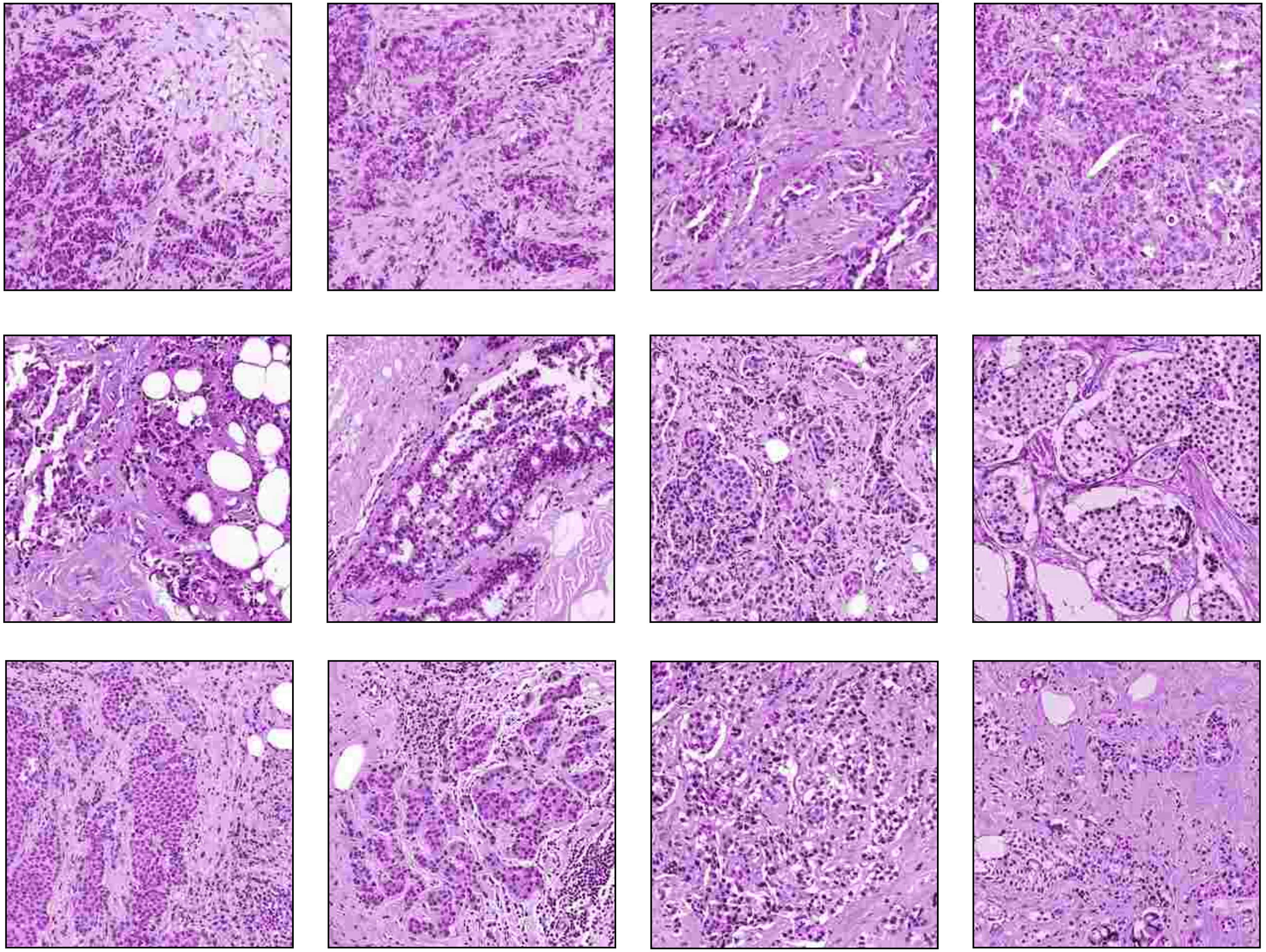

10x Cancer Monochrome 1:27 JPEG Set B

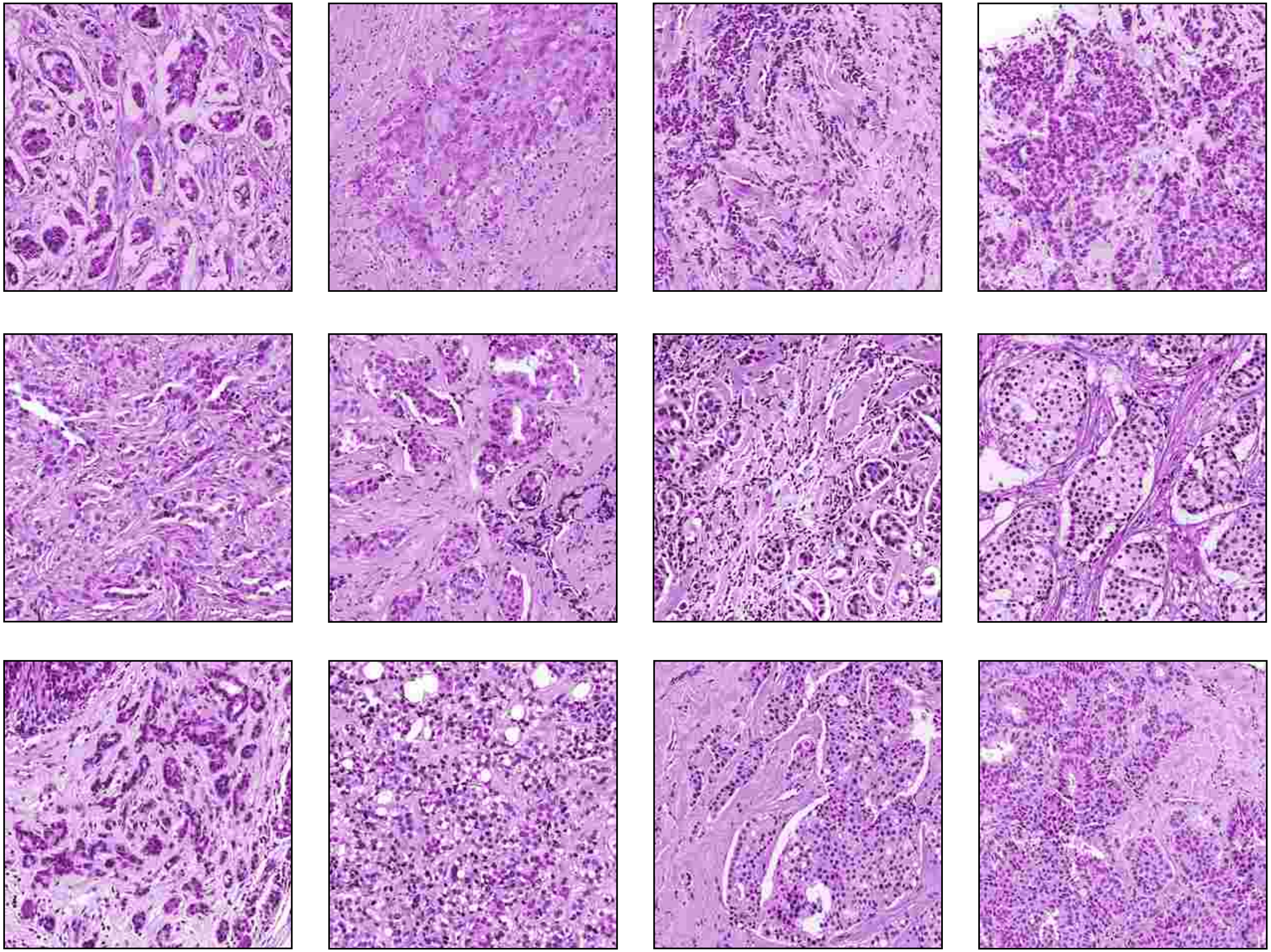

Supplement: S1 File — (PDF) [file pone.0141357.s001.pdf]
