# Supplementary material for: Pigeons (Columba livia) as Trainable Observers of Pathology and Radiology Breast Cancer Images
Source: PLoS One. 2015 Nov 18;10(11):e0141357. doi: 10.1371/journal.pone.0141357 (PMC4651348; doi:10.1371/journal.pone.0141357)

Microcalcifications (-) Set A

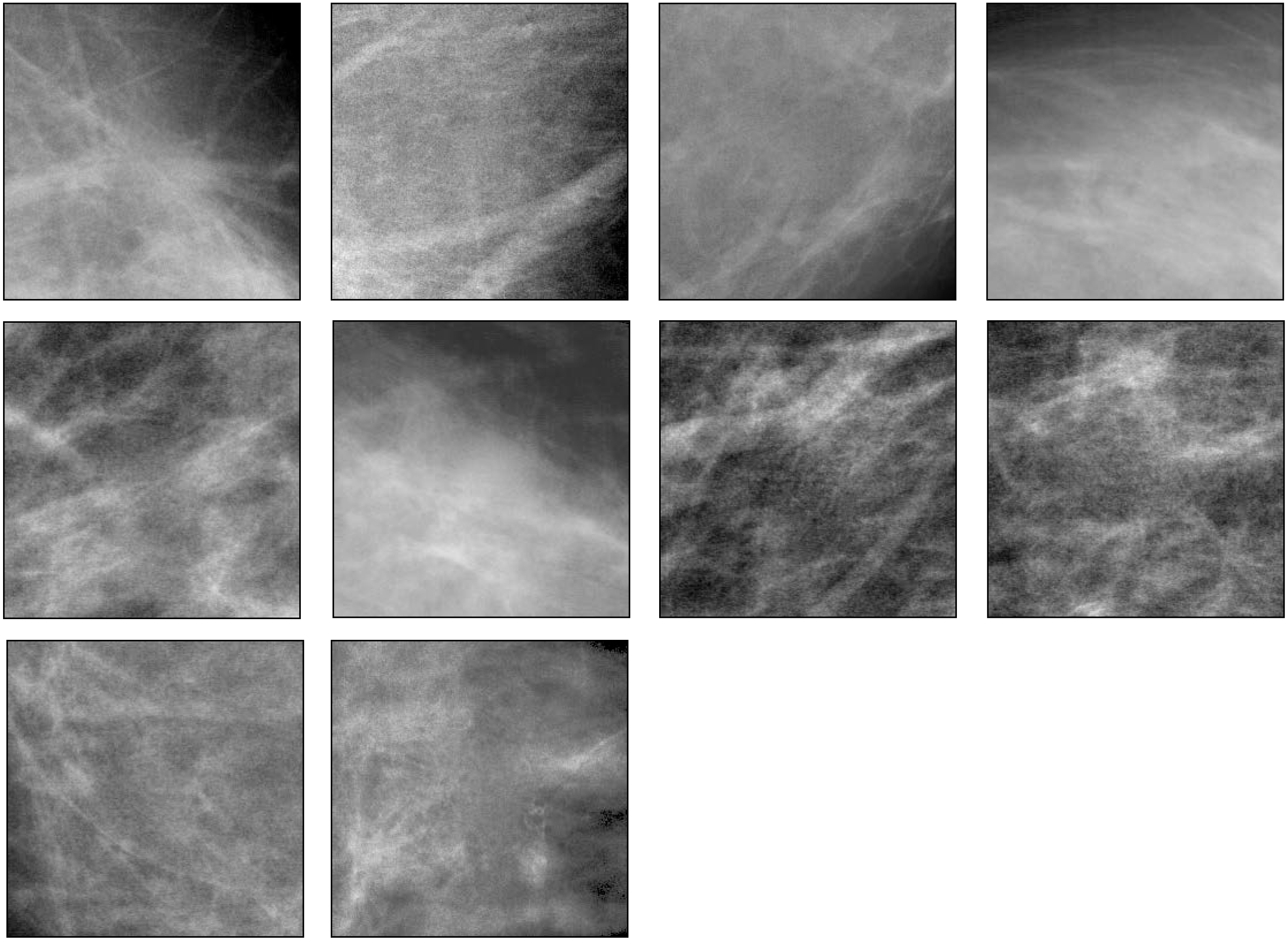

Microcalcifications (-) Set B

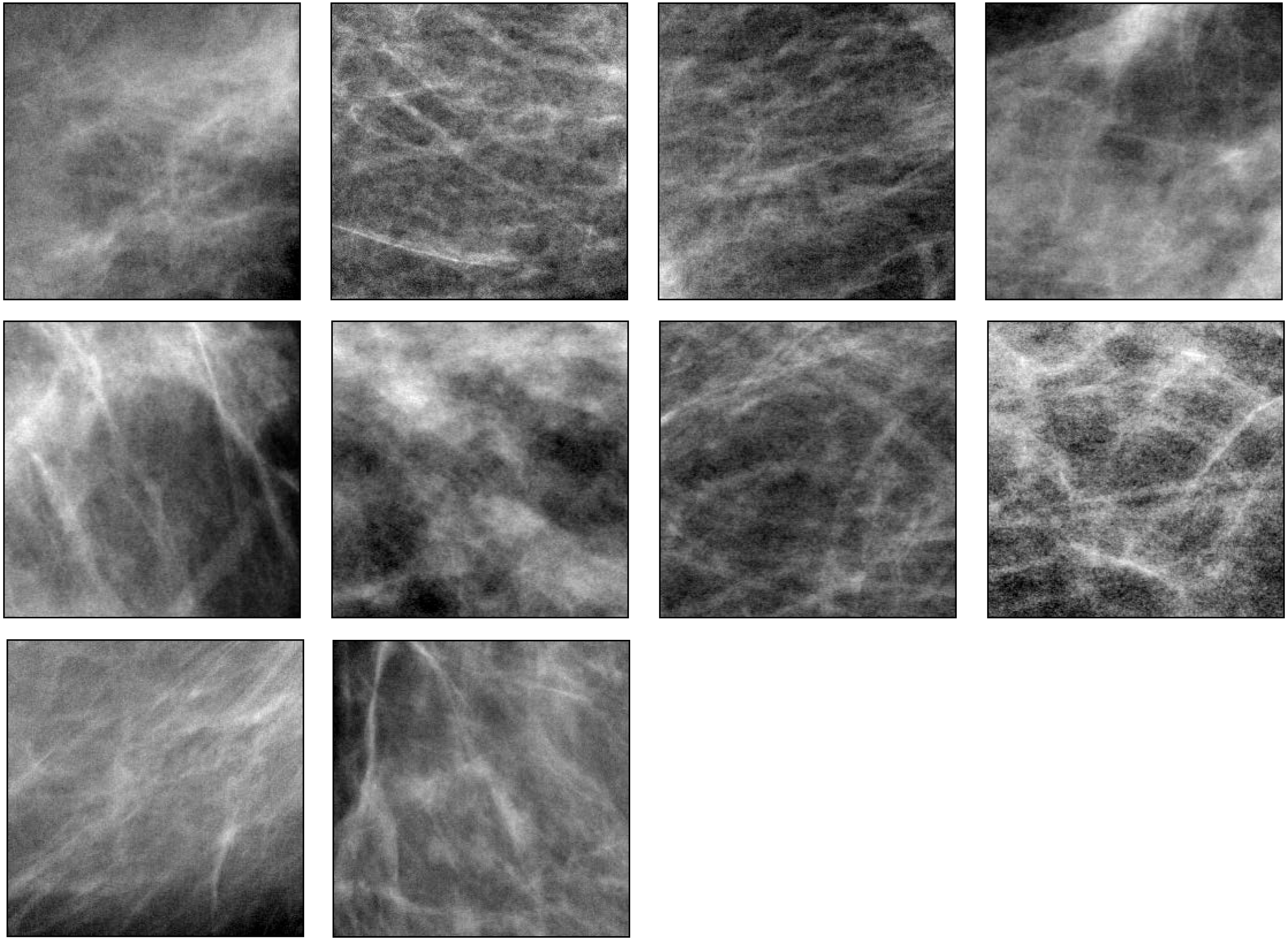

Microcalcifications (+) Set A

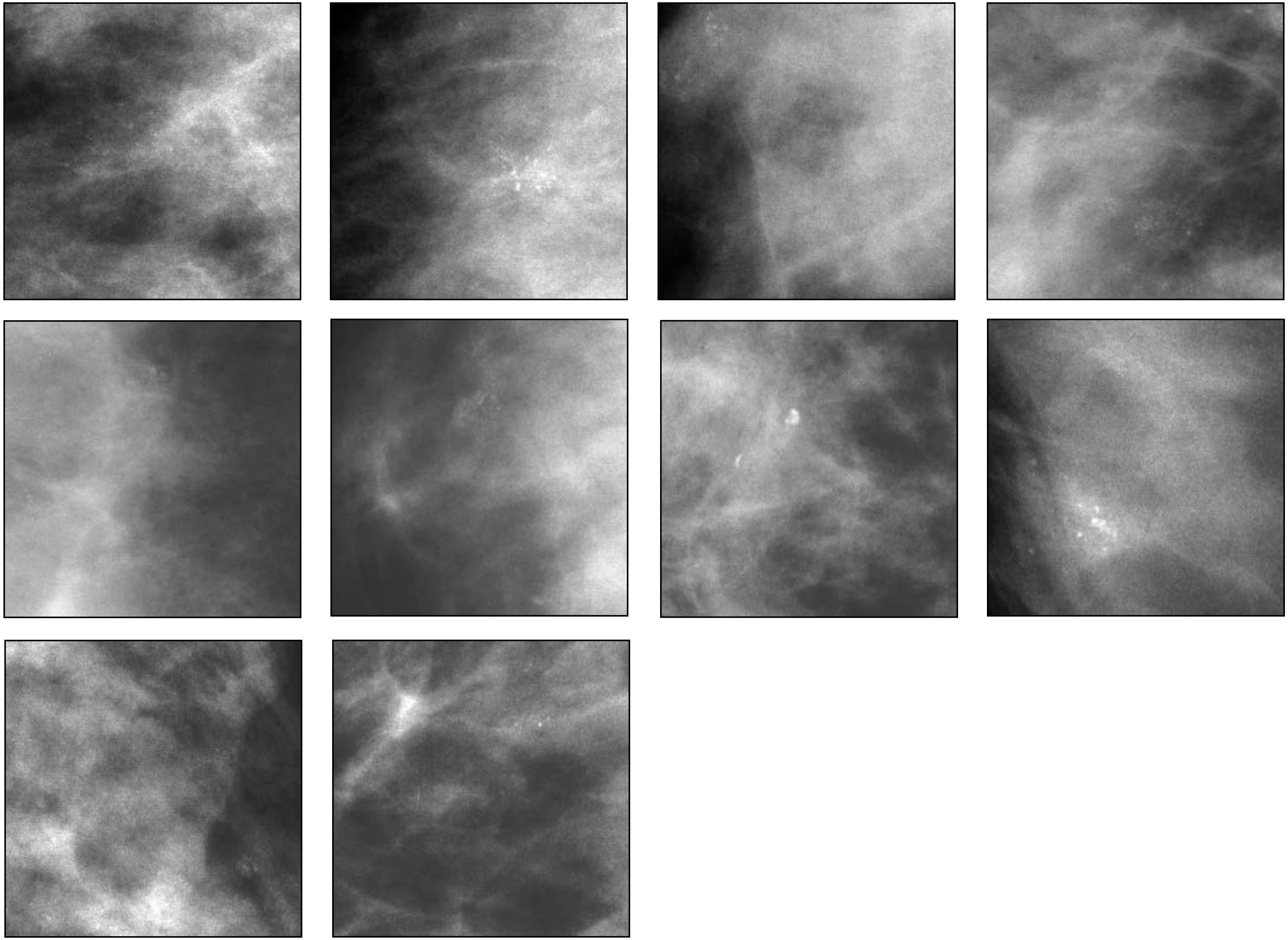

Microcalcifications (+) Set B

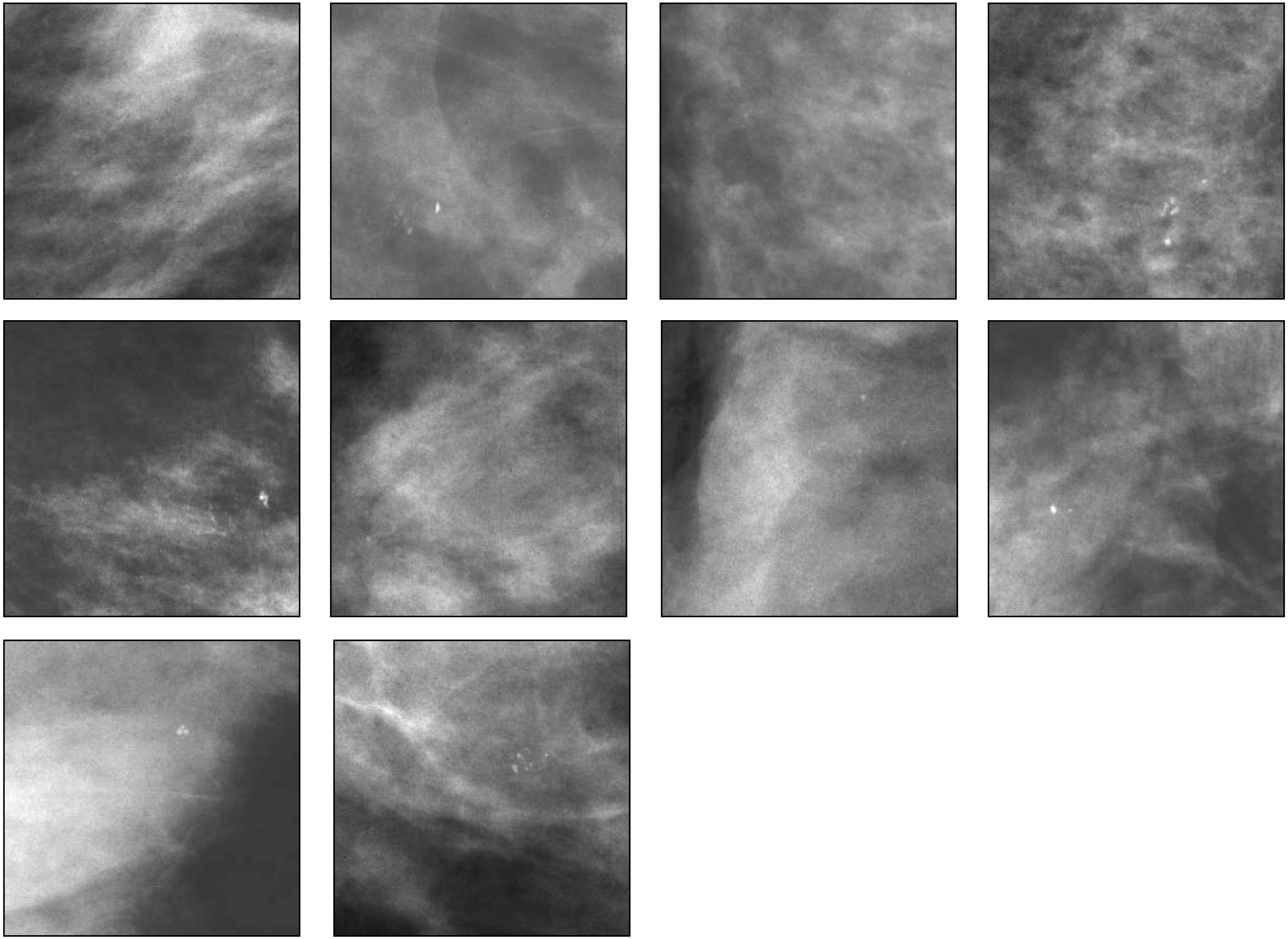

Mammogram masses, benign--Set A

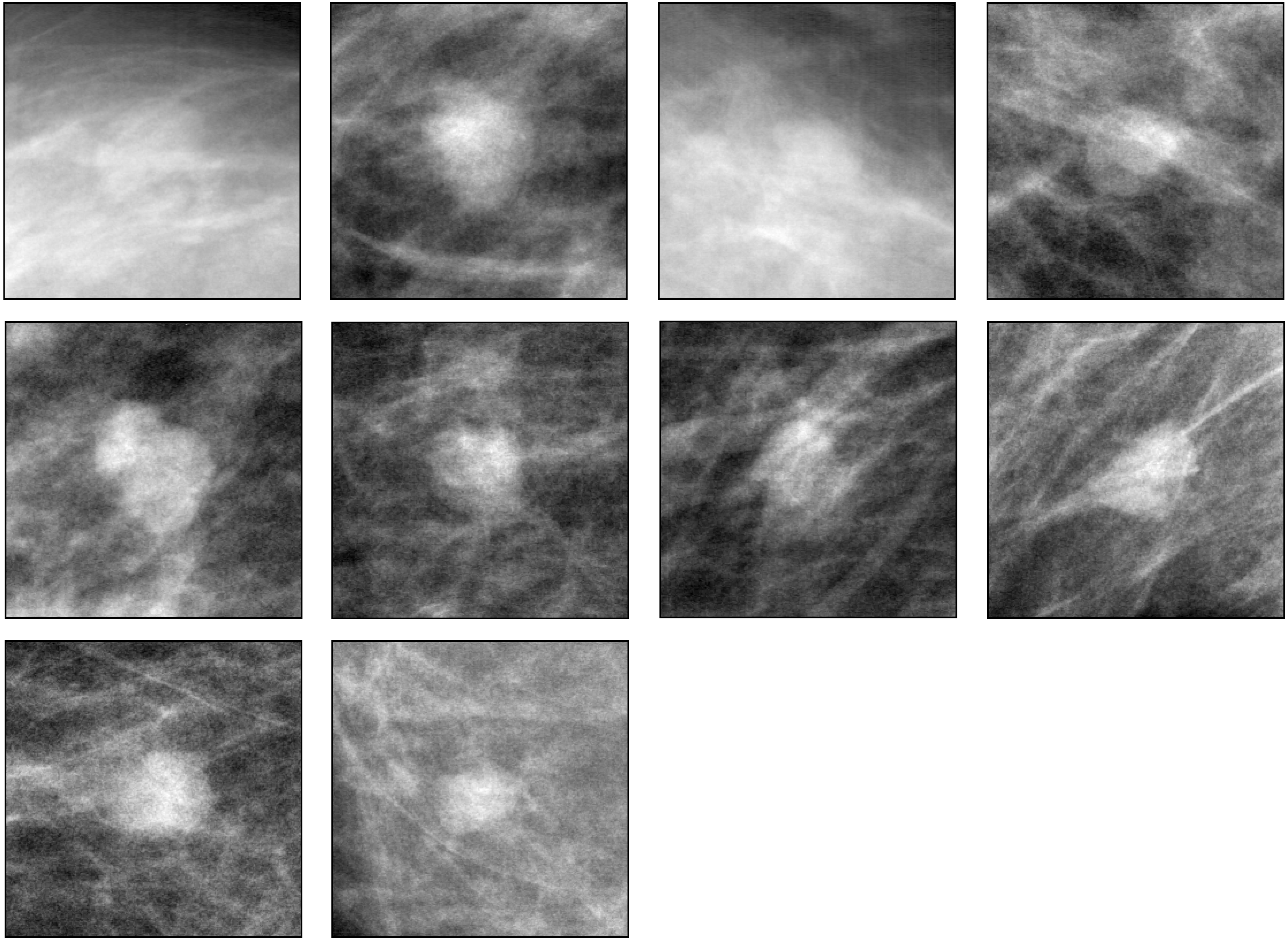

Mammogram masses, benign--Set B

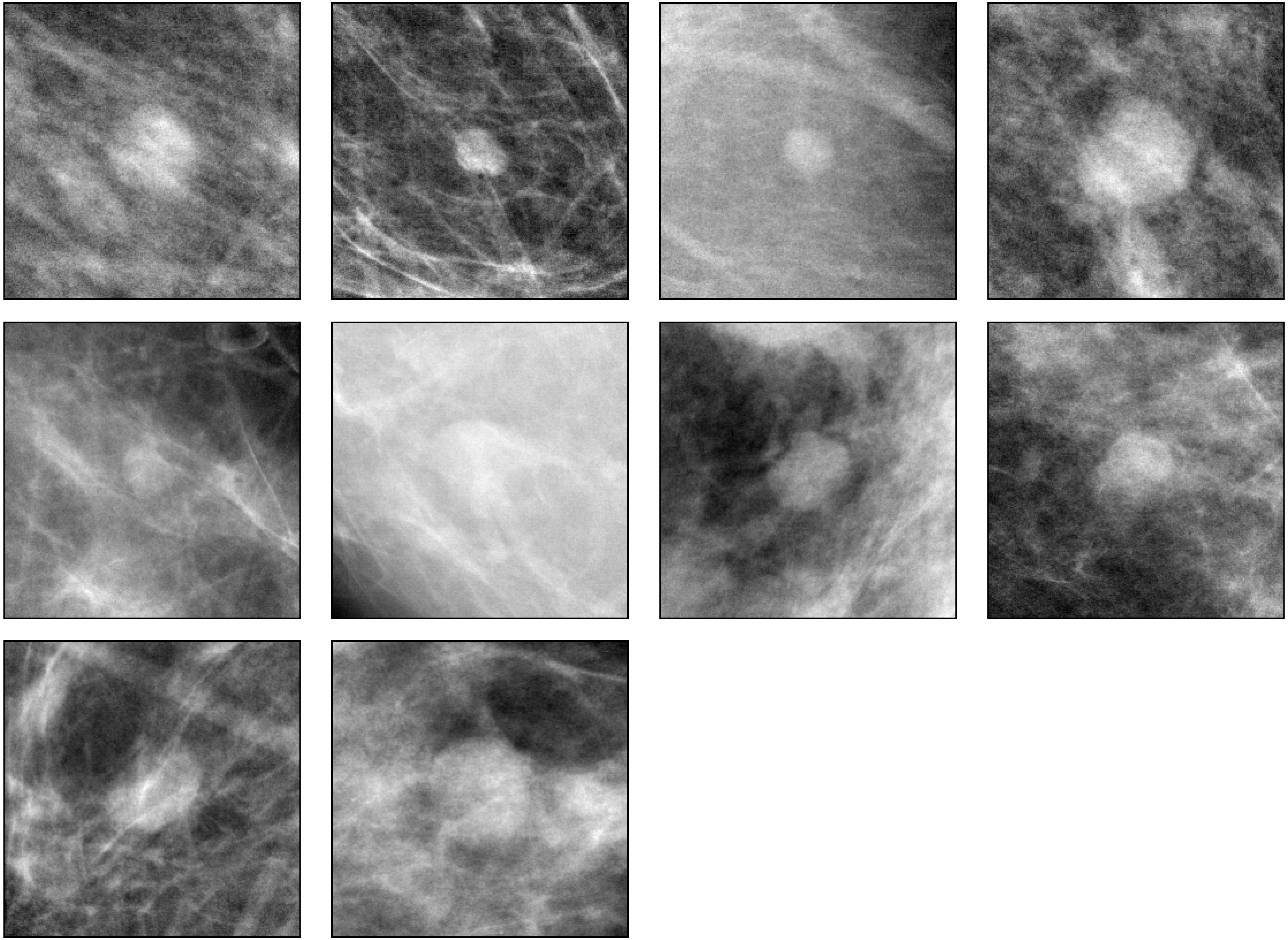

Mammogram masses, malignant--Set A

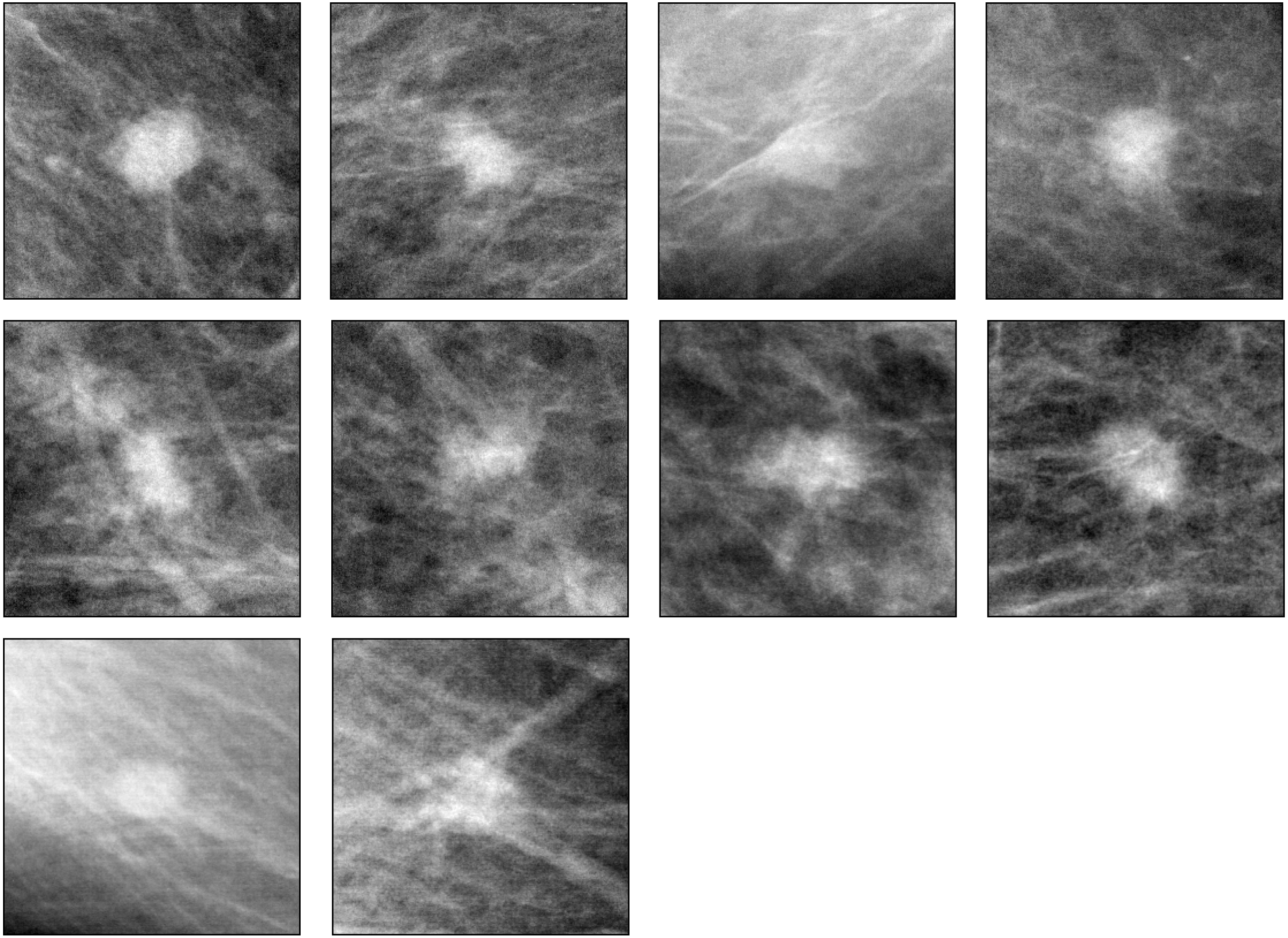

Mammogram masses, malignant--Set B

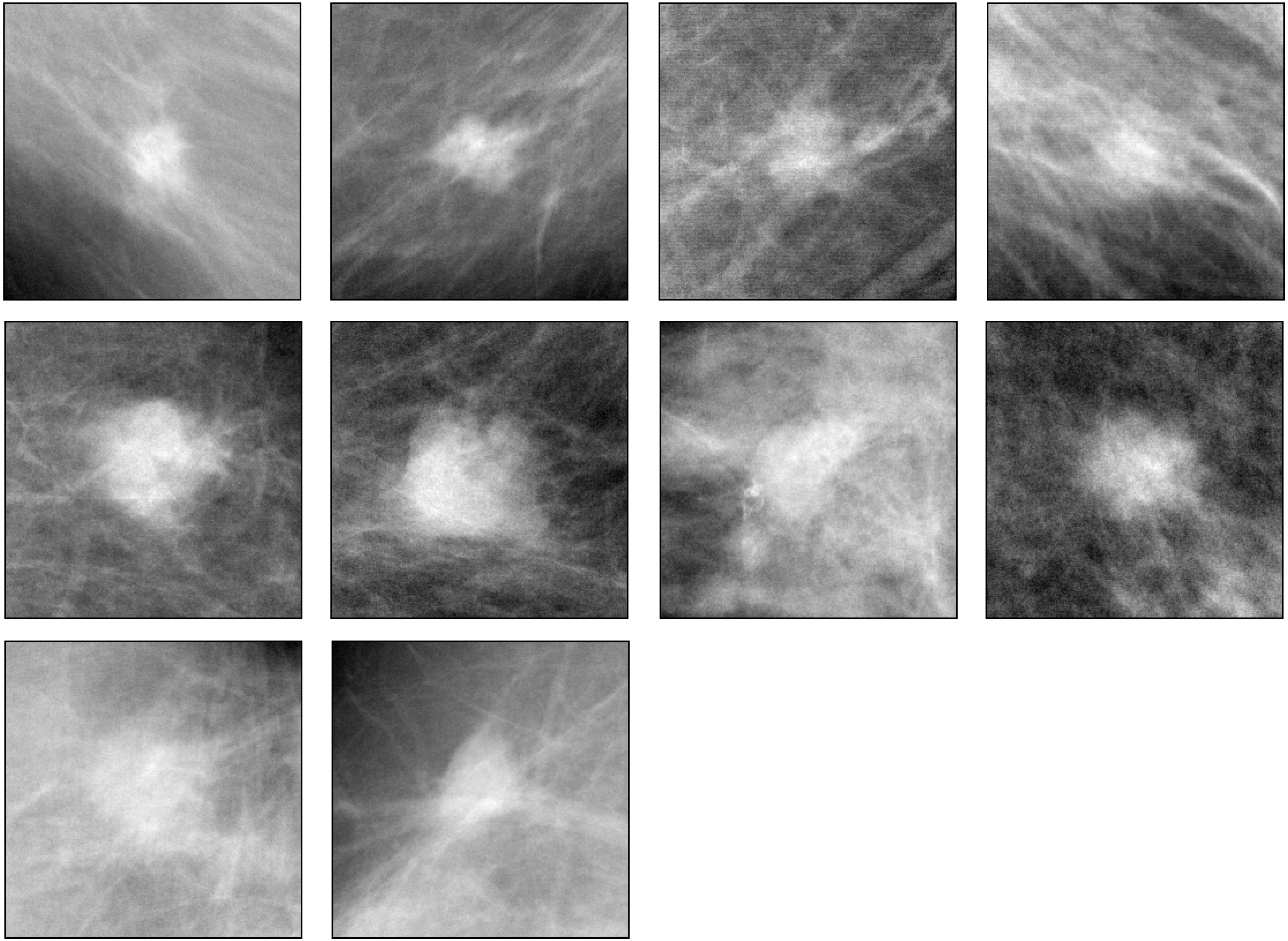

Supplement: S2 File — (PDF) [file pone.0141357.s002.pdf]
