# Supplementary material for: Pigeons (Columba livia) as Trainable Observers of Pathology and Radiology Breast Cancer Images
Source: PLoS One. 2015 Nov 18;10(11):e0141357. doi: 10.1371/journal.pone.0141357 (PMC4651348; doi:10.1371/journal.pone.0141357)
Supplement: S3 File — (ZIP) [file pone.0141357.s003.zip › S3 File - Experimental data/readme.docx]

This readme file contains information regarding the files that compose the experimental data of “Pigeons (*Columba livia*) as Trainable Observers of Pathology and Radiology Breast Cancer Images”

File name: exp1a.csv

Data format: Wide.

Description: Data for histology samples at different magnifications.

Naming scheme:

- ID codes the subject name.
- The magnification levels are coded as 4X, 10X, and 20X.
- The Training with 0° orientation stimuli and Training with rotated stimuli are coded in the header as TRAIN and TRAIN2, respectively.
- During rotation training (TRAIN2), scores with 0° orientation stimuli are coded as R0, whereas scores with rotated stimuli are coded as RALL.
- Testing phases are coded under the name GENTEST. The type of testing was coded numerically: odd numbers correspond to novel stimuli testing (1 for 4x, 3 for 10x, and 5 for 20x), whereas even numbers correspond to higher magnification testing (2 for 4x-10x, and 4 for 10x-20x).
- Regardless of the type of test, scores with the training stimuli are coded as OLD, whereas scores with the novel stimuli are coded as NEW.
- Sessions are coded at the end of the header name (e.g. the third training session with 4x magnification stimuli is called TRAIN_4X_3).

File name: exp1b.csv

Data format: Wide.

Description: Data for histology samples that were hue-brightness equated.

Naming scheme:

- ID codes the subject name.
- Training with 0° orientation stimuli only is coded as TRAIN, and training with the expanded set of rotated images has the added suffixes R0, for scores with 0° orientation stimuli, or RALL, for scores with rotated stimuli.
- The testing phase is coded under the name GEN. Scores with the training stimuli are coded as OLD, whereas scores with the novel stimuli are coded as NEW.
- The interposed training with familiar and novel sets was coded as A for the training set, and B for the novel set. The training with both sets mixed is coded as M.
- Sessions are coded at the end of the header name (e.g. the third training session with the mixed sets is called M_3).

File name: exp1c.csv

Data format: Wide.

Description: Data for histology samples that were compressed.

Naming scheme:

- ID codes the subject name.
- Testing with the compressed stimuli was coded TEST, whereas training with compressed stimuli was coded TRAIN.
- The compression levels were coded NOCOMP, COMP30, and COMP10 for uncompressed, 7% compression, and 1% compression stimuli.
- Sessions are coded at the end of the header name (e.g. the third training session with 7% compression stimuli is called TRAIN_COMP30_3).

File name: exp1b-roc.txt

Data format: Long.

Description: Data used for the ROC analyses.

Naming scheme:

- Source refers to the source of the judgment. It can be any of the individual birds (28Y, 29B, 45W, or 71R), Flock (for the flock sourcing score), or State (to denote the actual state of the stimulus).
- Stimulus denotes the stimulus number.
- Category denotes the stimulus category. Malignant slides were coded as M, whereas benign slides were coded as B.
- Judgement has a different meaning depending on the source. For individual birds codes the type of response the birds made when presented the slide. A benign response was coded as 0, whereas a malignant response was coded as 1. For the flock, this variable represents the sum of the individual birds’ responses to the given slide. Finally, when the source is State, this variable codes for the actual category of the slide (0 for benign, and 1 for malignant).

File name: exp2.csv

Data format: Wide.

Description: Data for mammograms with microcalcifications.

Naming scheme:

- ID codes the subject name.
- Training with 0° orientation stimuli only is coded as TRAIN, and training with the expanded set of rotated images has the added suffixes R0, for scores with 0° orientation stimuli or RALL, for scores with rotated stimuli.
- The testing phase is coded under the name TEST. Scores with the training stimuli are coded as OLD, whereas scores with the novel stimuli are coded as NEW.
- Sessions are coded at the end of the header name (e.g. the score for novel stimuli during the third testing session is called TEST_NEW_3).

File name: exp3.csv

Data format: Wide.

Description: Data for mammograms with masses.

Naming scheme:

- ID codes the subject name.
- Training is coded as TRAIN, whereas the testing phase is coded as TEST. Scores with the training stimuli are coded as OLD, whereas scores with the novel stimuli are coded as NEW.
- Sessions are coded at the end of the header name (e.g. the score for familiar stimuli during the third testing session is called TEST_OLD_3).
